# Organic Donor‐Acceptor Systems for Photocatalysis

**DOI:** 10.1002/advs.202307227

**Published:** 2023-12-25

**Authors:** Lingsong Wang, Weigang Zhu

**Affiliations:** ^1^ Key Laboratory of Organic Integrated Circuits Ministry of Education Tianjin Key Laboratory of Molecular Optoelectronic Sciences Department of Chemistry School of Science Tianjin University Tianjin 300072 China

**Keywords:** D‐A interactions, exciton dissociation, organic semiconductors, photocatalysis

## Abstract

Organic semiconductor materials are considered to be promising photocatalysts due to their excellent light absorption by chromophores, easy molecular structure tuning, and solution‐processable properties. In particular, donor‐acceptor (D‐A) type organic photocatalytic materials synthesized by introducing D and A units intra‐ or intermolecularly, have made great progress in photocatalytic studies. More and more studies have demonstrated that the D‐A type organic photocatalytic materials combine effective carrier separation, tunable bandgap, and sensitive optoelectronic response, and are considered to be an effective strategy for enhancing light absorption, improving exciton dissociation, and optimizing carrier transport. This review provides a thorough overview of D‐A strategies aimed at optimizing the photocatalytic performance of organic semiconductors. Initially, essential methods for modifying organic photocatalytic materials, such as interface engineering, crystal engineering, and interaction modulation, are briefly discussed. Subsequently, the review delves into various organic photocatalytic materials based on intramolecular and intermolecular D‐A interactions, encompassing small molecules, conjugated polymers, crystalline polymers, supramolecules, and organic heterojunctions. Meanwhile, the energy band structures, exciton dynamics, and redox‐active sites of D‐A type organic photocatalytic materials under different bonding modes are discussed. Finally, the review highlights the advanced applications of organic photocatalystsand outlines prospective challenges and opportunities.

## Graphical abstract

This review highlights recent advances in the field of donor‐acceptor (D‐A) organic photocatalysis. It covers a wide range of subjects, from intramolecular and intermolecular D‐A interactions between building blocks at the microscopic level to macroscopic functional applications. This review can serve as a comprehensive understanding of the design of next‐generation D‐A organic photocatalysts.

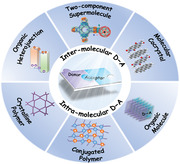

## 1 Introduction

Clean energy and environmental protection are necessary guarantees for the sustainable coexistence of all species on earth and need to be given sufficient attention. Photocatalytic technology has emerged as a cost‐effective and sustainable method in current research.^[^ ^1^, ^2^, ^3^ ^]^ It enables photocatalytic redox reactions to harness solar energy for generating hydrogen and hydrocarbons, such as water photolysis for hydrogen evolution, nitrogen fixation, and carbon dioxide reduction.^[^ ^4^, ^5^, ^6^ ^]^ Additionally, photocatalysis can effectively eliminate toxic environmental pollutants, as well as deactivate disease‐causing bacteria and tumors, thus safeguarding life and health.^[^ ^7^, ^8^, ^9^ ^]^ Consequently, solar energy‐based photocatalytic strategies hold significant promise in mitigating the prevailing energy and environmental crises.^[^ ^10^, ^11^ ^]^ Since the discovery of titanium dioxide photocatalysis for hydrogen evolution by Fujishima and Honda in 1972,^[^ ^12^ ^]^ researchers have been diligently exploring various aspects such as spatial configuration, electronic structure, interfacial reactions, and the theory of ultrafast charge dynamics.^[^ ^13^, ^14^ ^]^ The main objective of these efforts is to enhance the solar energy conversion efficiency of photocatalytic materials.^[^ ^15^ ^]^ The inorganic photocatalytic materials experience sufficient development. For instance, chalcogenides (e.g., Bi_2_S_3_, CdS, MoS_2_),^[^ ^16^, ^17^ ^]^ metal oxides (e.g., TiO_2_, Fe_2_O_3_, WO_3_),^[^ ^18^, ^19^, ^20^ ^]^ and composite metal oxides (e.g., BiVO_4_, BiOX, MnFe_2_O_4_).^[^ ^21^, ^22^ ^]^ In addition to physical and chemical stability, they also combine high dielectric constants and excellent carrier mobility.^[^ ^23^ ^]^ Of course, inorganic photocatalytic materials are limited by a narrow spectral response.^[^ ^24^, ^25^ ^]^

Organic semiconductors are mainly characterized by monocyclic or polycyclic closed systems of aromatic structure, with highly delocalized π‐electrons, low system energies, and greater stability.^[^ ^26^ ^]^ Organic semiconductors have garnered significant attention and research interest as a parallel alternative due to their unique properties. Comprising earth‐abundant elements such as carbon, hydrogen, nitrogen, and oxygen, at an affordable cost of preparation.^[^ ^27^ ^]^ Organic materials combine a wealth of isomeric (tectonic isomerism and stereoisomerism), electronic (charge transport and energy band structure), mechanical (flexible, stretchable), and optical (tunable emission and lifetime) properties.^[^ ^28^ ^]^ The inherent flexibility of these materials not only grants them improved solubility and easier preparation on a large scale for processing but also enables a greater tunability of electronic structures, expanding their responsiveness to a broader range of solar light, thereby making them favorable choices as organic photocatalysts.^[^ ^29^ ^]^ Furthermore, their rich variety of bonding modes, interaction types, and stacking patterns result in diverse electrical and optical behaviors, facilitating the establishment of correlations between organic molecular structure and photocatalytic performance.

Nevertheless, compared to inorganic semiconductors, organic semiconductors exhibit a smaller Frenkel exciton radius (≈5 Å) and a larger exciton Coulomb binding energy (0.3–1.0 eV), restricting the separation of e^−^‐h^+^ pairs within the same molecule and consequently leading to a constrained capacity for spontaneous dissociation into free charge carriers.^[^ ^30^ ^]^ This intrinsic limitation unavoidably impedes their photonic quantum efficiency. Many improvement strategies have been successively proposed to overcome the intrinsic defects of organic materials, for example, the most well‐known crystal engineering, interface engineering, and interaction modulation, and some exciting results can be seen.

Crystal engineering is proven to be a potent method to improve the optical, electronic, and photocatalytic properties of organic materials. The morphological attributes, such as shape, size, crystalline quality, and stacking arrangement of organic crystals, play a crucial role in dictating their extrinsic photoelectronic properties.^[^ ^31^ ^]^ Specifically, undesirable factors encompassing disordered alignments, extensive crystal dimensions, challenging‐to‐remedy defects, and crystal boundaries collectively exert notable impacts on light absorption and charge mobility, and can even induce energetic and structural perturbations.^[^ ^32^ ^]^ In this perspective, precise control of the thermodynamic and kinetic conditions of crystal growth is essential to modulate charge transport and migration. The “ice‐melting” strategy for organic crystal growth has been proposed by Wang et al. to optimize the photocatalytic performance of organic nanocrystals at the kinetic level.^[^ ^33^ ^]^ The ice‐melting of organic molecules from a relaxed environment allowed optimization of the nucleation and growth conditions by slowing down the reaction kinetics and increasing the energy barriers to obtaining well‐filled nanocrystals. Bai et al. have achieved the control of crystal morphology using surfactants,^[^ ^34^ ^]^ and under using cetyltrimethylammonium bromide (CTAB), tin porphyrin crystallized from octahedral nanocrystals, which shows better photocatalytic hydrogen evolution performance. This is attributed to the fact that CTAB facilitated non‐covalent interaction forces between molecules, which has demonstrated the dependence on molecular stacking, crystal engineering, and photocatalytic performance.

Interface engineering has gained significant traction in the realm of inorganic photocatalytic materials, and it has emerged as a promising approach for the modification of organic semiconductors as well.^[^ ^35^ ^]^ Abundant studies have explored the development of type‐II, type‐III, type‐Z, and type‐S organic heterojunctions,^[^ ^36^, ^37^, ^38^ ^]^ aiming to facilitate effective exciton dissociation and charge transfer, thereby substantially enhancing photocatalytic performance. Among them, type II heterojunction is efficient in the separation of charge carriers, but both electrons and holes are moved to lower energy levels, resulting in a weakened redox capacity.^[^ ^39^ ^]^ Z‐type heterojunction is a simulation of the charge transfer mode applied in the photosynthesis stage of plants, which effectively improves the redox capacity of photocatalysts. However, additional redox mediators are required to be introduced, leading to unforeseen side reactions.^[^ ^40^, ^41^ ^]^ In recent years, the S‐type heterojunction proposed by Yu et al. is mainly constructed from a reduced semiconductor with a small work function and a high fermi energy level and an oxidized semiconductor with a large work function and a low fermi energy level, which can realize the effective separation of e^−^‐h^+^ pairs. However, the requirement of materials with suitable energy band structures and significant fermi energy level differences limits the choice of photocatalysts greatly.^[^ ^38^, ^42^ ^]^ In addition, functionalized organic semiconductors can be compatible with inorganic materials to construct organic‐inorganic heterojunctions, capitalizing on the superior light‐absorbing capabilities of organic chromophores and the high mobility exhibited by inorganic counterparts, resulting in remarkably efficient photoelectric and photochemical conversion efficiencies.^[^ ^43^, ^44^ ^]^ The other option is a combination with a co‐catalyst to provide active sites and facilitate e^−^‐h^+^ separation. The co‐catalysts are usually divided into noble metals (e.g., Pt, Pd, Ru, Rh, Ir),^[^ ^45^, ^46^ ^]^ and transition metal oxides (e.g., RuO*_n_*, IrO*_n_*, MnO*_n_*, CoO*_n_*).^[^ ^47^, ^48^ ^]^ The paramount significance of interface engineering in organic semiconductors warrants diligent scrutiny and profound investigation, yet regrettably, detailed discourse on this subject is constrained by the limitations of the present capacity. Of course, there are many excellent research teams that have comprehensively reviewed interface engineering in organic semiconductors,^[^ ^49^, ^50^, ^51^ ^]^ rendering their work highly instructive and enlightening for further scholarly exploration.

The intricate and interwoven interactions in organic materials play a significant role in shaping their external performance, presenting a captivating challenge for researchers to unravel. In addition to π–π stacking as the main driving force, other types of interactions can affect and even change the intrinsic properties of organic materials, such as dipole‐dipole interactions, spatial effects of end‐groups, donor‐acceptor (D‐A) interactions, and so on.^[^ ^52^, ^53^ ^]^ D‐A materials, comprising electron‐rich donor units and electron‐deficient acceptor units linked by covalent or non‐covalent bonds, have emerged as crucial elements in diverse fields such as luminescence regulation, solar cells, photoelectric conversion, and photocatalysis.^[^ ^54^, ^55^ ^]^ The large dipole moment in the D‐A material is due to the disparate electron affinity potentials between the donor and acceptor, thereby facilitating charge transfer (CT) from the D to A in the excited state.^[^ ^56^ ^]^ Ultimately, the photogenerated electrons are advantageously concentrated on the acceptor molecule, which can efficiently facilitate the exciton dissociation. In addition, CT interactions and the increase of π‐conjugated fragments narrow the material band gap effectively, yielding a redshift of the optical absorption edge and widening the solar spectral response range. Therefore, intermolecular D‐A or intramolecular D‐A structures are considered effective strategies for the enhancement of optical absorption, improvement of exciton dissociation, and optimization of carrier transport.^[^ ^57^ ^]^

**Figure 1:**
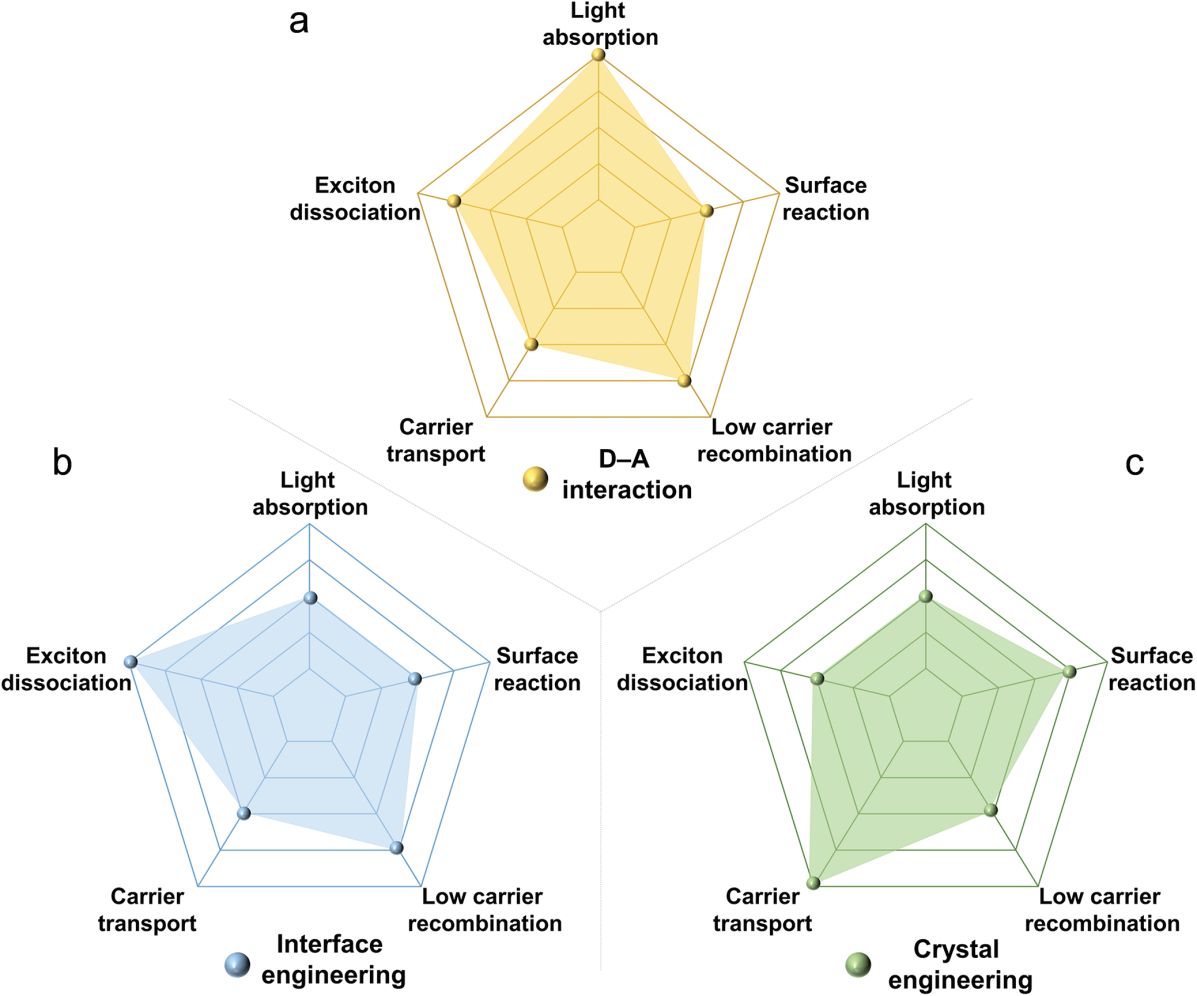
Schematic illustration of the differences between the three modification methods from the perspective of photocatalytic elementary processes. a) D‐A interactions. b) Interface engineering. c) Crystal engineering.

Interface engineering, crystal engineering, and interaction modulation strategies, with a primary focus on D‐A interactions, have been analyzed to enhance the photocatalytic properties of organic materials. Each of these strategies plays a distinctive role in five fundamental aspects: broadening spectral absorption, promoting exciton dissociation, accelerating photogenerated carrier transport, inhibiting charge recombination, and facilitating surface photocatalytic reactions. As shown in **Figure** **1**, different strategies can be chosen by researchers to enhance the performance of organic photocatalytic materials, or utilizing a combination of different strategies to achieve the same purpose. Moreover, it is certainly more creative to design and develop more novel and effective solutions for enhancing photocatalytic performance.

In this review, we present a focused analysis of D‐A interactions and provide a comprehensive overview of D‐A organic photocatalysts, including conjugated polymers, crystalline polymers (COFs, MOFs), organic small molecules, supramolecules, and organic heterojunction materials, along with a concise examination of their advanced applications in photocatalysis. D‐A organic photocatalysts have been extensively reported and their photocatalytic properties are gradually climbing up. However, to the best of our knowledge, there are only a few review articles focusing on the role of D‐A interactions in photocatalysis. By bridging this gap, our review aims to provide valuable references for people engaged in related studies and to actively stimulate greater engagement from more researchers in the advancement of D‐A organic photocatalysts.

## 2 Intramolecular D‐A Interactions

### 2.1 D‐A Type Organic Molecules

#### 2.1.1 Thiophene and Derivatives

**Figure 2:**
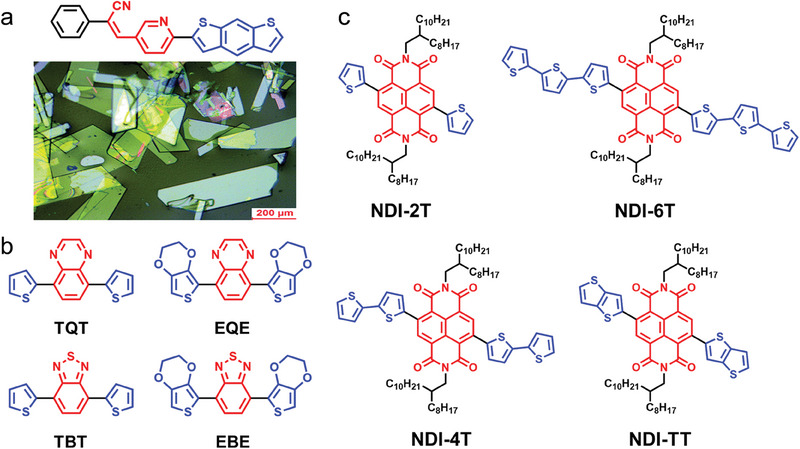
Molecular structure of thiophene and its derivatives with D‐A structure. a) The structure and microscopic images of the PCPyBDT molecule. Reproduced with permission.^[^ ^58^ ^]^ Copyright 2021, Royal Society of Chemistry. b) Four D‐A‐D conjugated molecular structures with different D and A units. c) Molecular structures with different numbers of thiophene units based on NDI acceptor.

The 6π electrons system formed by the sulfur atom and four carbon atoms together in the thiophene molecule exhibits electron‐giving properties. A new conjugated organic molecule PCPyBDT has been constructed with benzo[1,2‐*b*:5,4‐*b'*]dithiophene (BDT) as the donor unit and pyridine, cyano‐group as the acceptor unit and prepared as sheet crystals by Ma et al.^[^ ^58^ ^]^ The efficiency of PCPyBDT for optical capture and utilization is enhanced due to the translucent nature and highly crystalline surface of the nanosheets (**Figure** **2**a). In addition, high electron transport rates are generated by strong intramolecular D‐A interactions and compact intermolecular stacking arrangements, which exhibit good electron mobility of 0.25 cm^2^ V^−1^ s^−1^ and an excellent hydrogen evolution rate of 8143 µmol g^−1^ h^−1^. Eric Cloutet and coworkers investigate the role of different D and A units on the photophysical and photocatalytic properties of D‐A‐D conjugated trimers. As in Figure 2b, quinoxaline (Q) and benzothiadiazole (B) are chosen as A units, and 3,4‐ethylenedioxythiophene (E) and thiophene (T), both with strong electron‐donating ability, are used as D units, respectively.^[^ ^59^ ^]^ The D‐A‐D molecule (EBE) comprising E and B units performs the best photochemical performance with the photocatalytic hydrogen evolution rate of 142 µmol g^−1^ h^−1^. Naphthalene diimide (NDI) is a classical n‐type semiconductor with superior electron‐withdrawal and charge transport properties. Lai et al. have designed a D‐A‐D molecule with different numbers of thiophene units,^[^ ^60^ ^]^ namely NDI‐TT, NDI‐2T, NDI‐4T, and NDI‐6T, based on NDI (Figure 2c). Among them, the largest redshift absorption, the strongest e^−^‐h^+^ separation efficiency, and the longest exciton lifetime are reflected in the NDI‐6T molecule. The oxidation potential, however, is significantly weakened due to the excess of thiophene units, making the photocatalytic performance of NDI‐6T much lower. With the appropriate redox potential, the photocatalytic CO_2_ to CO conversion yield of NDI‐4T is 168.6 µmol g^−1^ 24 h^−1^.

#### 2.1.2 Carbazole and Derivatives

**Figure 3:**
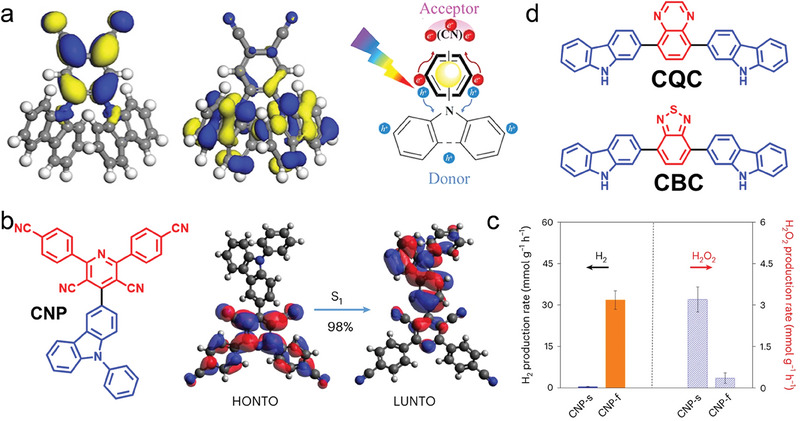
Molecular structure of carbazole and derivatives with D‐A structure. a) The structure of the 2CzPN molecule. Reproduced with permission.^[^ ^61^ ^]^ Copyright 2019, Wiley‐VCH. b) Chemical and electronic structure of CNP molecule. c) Photocatalytic properties of different aggregation state structures. Reproduced with permission.^[^ ^62^ ^]^ Copyright 2023, Springer Nature. d) Molecular structures with different A units based on carbazole donors.

Carbazole is an important nitrogen‐containing heterocyclic aromatic compound with a large conjugation system, which owns good photoelectric properties and thermal stability by itself and its derivatives. Carbazole‐cyano (2CzPN) supramolecular photocatalysts with a D‐A structure have been synthesized via a one‐step nucleophilic substitution reaction of carbazole and 4,5‐difluorophthalonitrile,^[^ ^61^ ^]^ and the structure of 2CzPN is shown in **Figure** **3**a. The strong D‐A interactions and high crystallinity of 2CzPN supramolecules compared to the polymeric state enable them to exhibit better photocatalytic activity than the polymers, although the BET surface area is much lower. Recently, Cooper et al. have reported a new organic molecule 2,6‐bis(4‐cyanophenyl)‐4‐(9‐phenyl‐9H‐carbazol‐3‐yl)pyridine‐3,5‐dicarbonitrile (CNP) with D‐A structure (Figure 3b), and the CNP exhibits two aggregated state morphologies of π–π ordered stacked nanofibrous (CNP‐f) and amorphous nanosphere (CNP‐s).^[^ ^62^ ^]^ As shown in Figure 3c, the rate of CNP‐f photocatalytic hydrogen evolution reaches up to 31.85 mmol g^−1^ h^−1^, while CNP‐s photocatalytic hydrogen evolution is very low but with excellent H_2_O_2_ production (3.20 mmol g^−1^ h^−1^ in the presence of O_2_). The different photocatalytic behaviors of the two aggregates are caused by the differences in excited state kinetics, the degree of excited state delocalization, and the rate of charge transfer to O_2_, and these differences can be attributed to the variation in molecular stacking in CNP‐f and CNP‐s. This study highlights the effect of molecular stacking in the aggregated state, and molecular crystalline structure on photocatalytic activity. Eric Cloutet et al. also constructed a D‐A‐D conjugated molecule using carbazole (C) as the D unit and quinoxaline (Q) and benzothiadiazole (B) as the A unit.^[^ ^59^ ^]^ As in Figure 3c, the CBC and CQC trimers show stable photocatalytic hydrogen evolution performance.

#### 2.1.3 Diazole Derivatives

**Figure 4:**
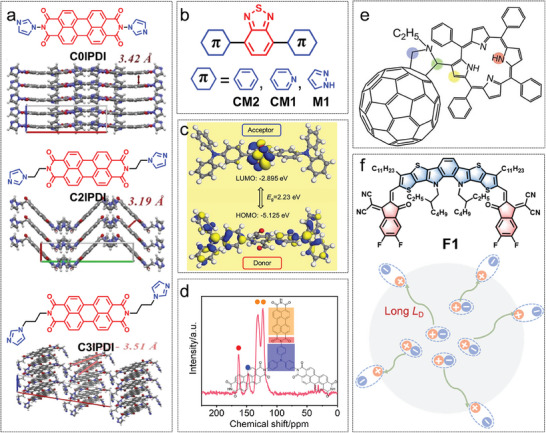
Molecular structures of diazole derivatives and other donor types with D‐A structures. a) Chemical and crystal structures of D‐A molecules based on imidazole and PDI. Reproduced with permission.^[^ ^63^ ^]^ Copyright 2023, Wiley‐VCH. b) Chemical structures of D‐A molecules containing different numbers of nitrogen atoms. c) Energy level structure of BDTD molecule based on triphenylamine donor. Reproduced with permission.^[^ ^65^ ^]^ Copyright 2019, Elsevier. d) Chemical structure of a triphenylamine‐PDI (D‐A) molecule with crystallinity. Reproduced with permission.^[^ ^66^ ^]^ Copyright 2022, Wiley‐VCH. e) Chemical structure of the SA‐TPP‐C_60_ molecule. Reproduced with permission.^[^ ^67^ ^]^ Copyright 2022, Elsevier. f) Organic photovoltaic photocatalyst F1 molecules with D‐A units. Reproduced with permission.^[^ ^68^ ^]^ Copyright 2022, American Chemical Society.

As shown in **Figure** **4**a, Chen et al. have regulated the π–π stacking distance and molecular stacking pattern by controlling the dihedral angle and spatial site resistance between the two rings of perylene diimide (PDI) and imidazole (IMZ) in C*n*IPDI (*n* = 0, 2, 3).^[^ ^63^ ^]^ In comparison, the shortest π–π stacking distance (3.19 Å) and the herringbone stacking pattern allow 2D charge transport in the C2IPDI molecules due to the ethyl access, resulting in the fastest carrier transport rate. The photocatalytic O_2_ production rate of IMZ‐alkyl‐PDI is 271 times higher than that of IMZ‐PDI, and the phenol degradation rate has increased 32 times. Xu et al. have synthesized a series of nitrogen‐containing D‐A conjugated molecule photocatalysts by substituting benzene, pyridine, and pyrazole for the donor units to control the number of N atoms.^[^ ^64^ ^]^ As shown in Figure 4b, the photocatalytic properties of small molecules are gradually enhanced with the increase in the number of N atoms. The D‐A interaction is elevated due to the increase of N atoms in the molecular system and supplies N active sites for photocatalytic interfacial reactions. In addition, hydrogen bonds formed by N atoms and water are used to improve the water contact and dispersion of organic conjugated molecules.

#### 2.1.4 Other Types of Molecules

As shown in Figure 4c, You et al. have synthesized a novel conjugated D‐A photocatalytic molecule 4,4′'‐bis(diphenylamino)‐[1,1′:4′,1′'‐terphenyl]‐2′,5′‐dicarbaldehyde (BDTD),^[^ ^65^ ^]^ consisting of a benzene ring containing an aldehyde group and a triphenylamine rotated at an angle at both sides, displaying as a dark yellow powder. Under visible light irradiation, bromate (BrO_3_^−^) can be effectively degraded to bromide by BDTD supramolecule, showing excellent photocatalytic bromate reduction ability. High crystallinity triphenylamine‐PDI molecules are designed and synthesized by Zhu et al.^[^ ^66^ ^]^ for the selective photocatalytic oxidation of 1,2,3,4‐tetrahydroisoquinoline to 3,4‐dihydroisoquinoline with 92% selectivity. The enhanced catalytic activities owing to the formation of the D‐A structure (Figure 4d) by the introduction of the electron donor triphenylamine into the PDI, contribute greatly to the separation of the photogenerated charges.

A typical donor molecule, tetraphenylporphyrin, has been linked to fullerene (C_60_) through covalent bonds as a novel D‐A supramolecular photocatalyst.^[^ ^67^ ^]^ The structure of SA‐TPP‐C_60_ is shown in Figure 4e. A highly ordered D‐A interaction in the SA‐TPP‐C_60_ molecule results from the intramolecular dipole interaction, leading to the formation of a powerful built‐in electric field (IEF), and facilitating ultrafast and ultra‐long lifetime charge separation. Remarkably, the SA‐TPP‐C_60_ single molecule photocatalytic hydrogen evolution rate is 10.69 mmol g^−1^ h^−1^. The photovoltaic molecule is easy to process in solution, with an optimized molecular structure and an expandable light absorption range, and maintains a better hydrogen evolution rate to date. The A‐D‐A molecular structure was employed by Lin et al. to develop an organic photovoltaic molecular photocatalyst F1 with high photoluminescence quantum yield (PLQY, 9.3%).^[^ ^68^ ^]^ As shown in Figure 4f, the longer exciton diffusion length (20 nm) of the F1 molecule is obtained by cutting off the strong acceptor unit (the “thiadiazole” of Y6). Under the test conditions of AM 1.5 G, the photocatalytic hydrogen precipitation rate of F1 is as high as 152.60 mmol g^−1^ h^−1^, more than twice that of Y6.

At present, there are fewer studies on D‐A type small molecule photocatalytic materials, and their photocatalytic ability is still to be further improved. First, the relatively small π‐conjugation of D‐A type molecules limits the absorption of light. In contrast, PDI‐based molecules and photovoltaic molecules with a larger degree of conjugation can exhibit good performance, so expanding the degree of conjugation and maintaining a certain degree of redox capacity is key.^[^ ^69^ ^]^ Furthermore, the high crystallinity of the molecular materials favors carrier transport but likewise leads to enhanced hydrophobicity. Although hydrophilicity can be improved by functional group modification, it is still insufficient compared to porous organic materials. In addition, the rapid transport of carriers through covalent bonds in individual D‐A type molecules is relatively well‐defined. However, their aggregated states are usually bound to each other by noncovalent bonds, and the lack of direct bridges for carrier transport intermolecularly might cause the recombination of electrons and holes in the same molecule. The studies related to the performance and aggregated state structure of D‐A type small molecule photocatalysts are still relatively scarce.^[^ ^63^ ^]^ Therefore, researchers should maximize the utilization of the clear chemical and aggregated state structure of small molecules to analyze a series of physicochemical behaviors in the photocatalytic process.

### 2.2 D‐A Type Polymers

The charge separation and transport of the polymer system are strongly hindered because the structured building blocks have low dielectric constants, thus inhibiting the surface photocatalytic reaction. Polymer photocatalysts containing alternating compositions of electron donors and electron acceptors have been illuminated as a reliable solution to enhance photovoltaic and photocatalytic performance. Two commonly employed approaches for modifying D‐A polymers are as follows:^[^ ^70^ ^]^ 1) Modulation of the HOMO and LUMO energy levels of polymer materials by changing the donor or acceptor molecular structure; 2) Introduction of heteroatoms (such as nitrogen atoms, oxygen atoms, etc.) within the D‐A fragment to enhance the electronic properties of the polymer.

Recently, many successes have been achieved in photocatalysis by D‐A conjugated polymers with dibenzothiophene‐S, S‐dioxide (FSO), triazine derivatives, benzothiadiazole (BT), or other heterocycles containing sp^2^ hybridized nitrogen as acceptors. The vast diversity of organic molecule species and the inherent flexibility in D‐A pairing give rise to thousands of potential D‐A polymers. However, for the purpose of this review, we shall focus solely on D‐A polymers based on the three most classical acceptor molecules.

#### 2.2.1 Based on Dibenzothiophene‐S, S‐Dioxide (FSO)

**Figure 5:**
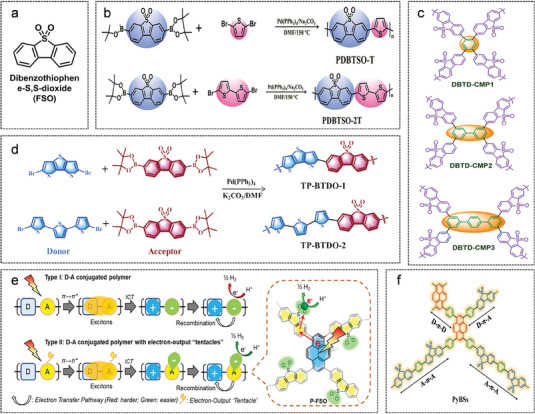
D‐A conjugated polymers based on FSO acceptor. a) The chemical structure of FSO. b) Diagram of the synthetic routes of polymers PDBTSO‐T and PDBTSO‐2T. Reproduced with permission.^[^ ^71^ ^]^ Copyright 2022, Elsevier. c) The chemical structure of D‐A polymers with different cross‐linker lengths. Reproduced with permission.^[^ ^72^ ^]^ Copyright 2018, American Chemical Society. d) D‐A polymers based on thiophene units with different degrees of conjugation. Reproduced with permission.^[^ ^73^ ^]^ Copyright 2022, Royal Society of Chemistry. e) Schematic diagram of the principle that the sulphonyl group in FSO acts as an electron‐output “tentacle”. Reproduced with permission.^[^ ^74^ ^]^ Copyright 2019, Elsevier. f) The D‐π‐A polymer strategy with π‐bridge. Reproduced with permission.^[^ ^75^ ^]^ Copyright 2021, Wiley‐VCH.

As shown in **Figure** **5**a, dibenzothiophene‐S, S‐dioxide (FSO) has been widely used as an acceptor unit in polymer photocatalysts because of its well‐conjugated structure, high π‐electron delocalization, and better hydrophilicity. Wang et al. have selected thiophene derivatives (thiophene and 2,2′‐dithiophene) with a six π‐electron, five‐membered planar structure as donors to provide the conjugate backbone with a certain electron density (Figure 5b).^[^ ^71^ ^]^ Two new D‐A conjugated polymers PDBTSO‐T and PDBTSO‐2T based on FSO are designed and synthesized, and PDBTSO‐T exhibits superior photocatalytic hydrogen evolution rate (107 mmol g^−1^ h^−1^) under natural sunlight irradiation. Theoretical calculations demonstrate that more thiophene units weaken the charge density of oxygen atoms in the sulphonyl group on the PDBTSO‐2T polymer and affect the hydrogen adsorption reduction process negatively. Jiang et al. have demonstrated that the photocatalytic performance of conjugated microporous polymers (CMPs) with FSO decreases with the increase of cross‐linker length.^[^ ^72^ ^]^ As shown in Figure 5c, with the replacement of the cross‐linking agent from benzene and biphenyl to *p*‐triphenyl in turn, the higher degree of distortion of the polymer backbone reduces the conjugation and planarity, thus hindering the transport and separation of photogenerated charges. Recently, two D‐A polymer photocatalysts with a wide optical absorption range were prepared in Jiang's group by polymerizing thiophene donors of a narrow band gap with FSO. The polymerization scheme is shown in Figure 5d.^[^ ^73^ ^]^ Compared with TP‐BTDO‐1, TPP‐BTDO‐2 containing 2,2′:5′,2″‐terthiophene shows stronger absorption of visible light and more effective separation of charge carriers.

Previous studies have demonstrated that the intrinsic exciton dissociation and carrier transport properties of benzo[1,2‐*b*:3,4‐*b′*:5,6‐*b″*]trithiophene (BTT) afforded it excellent optical energy conversion efficiency.^[^ ^76^ ^]^ Yao et al. have utilized BTT as donors for Suzuki coupling with FSO, BTT‐CPP polymers facilitate the separation and transport of charge carriers owing to the rigid planar structure and aromatic conjugation system of BTT. Thiophene‐contained polycyclic aromatic compounds as excellent donor units for D‐A polymer photocatalysts are demonstrated in this work. In 2015, Cooper's group reported visible light‐driven and bandgap tunable CMPs photocatalysts based on pyrene (Py) for the first time.^[^ ^77^ ^]^ Liu et al. use Py as donor building blocks to polymerize with FSO to obtain Py‐SO polymer with a hydrogen evolution rate of 4.74 mmol g^−1^ h^−1^.^[^ ^78^ ^]^ Reasonable structural polymerization by introducing polycyclic aromatic hydrocarbon molecules is a potential approach to constructing visible polymer photocatalysts. Wang's group has reported a series of Py‐based D‐A polymers that gradually replaced CP‐CMP10 with FSO.^[^ ^74^ ^]^ The performance of photocatalytic hydrogen evolution is gradually improved with the increase of FSO content in the polymer backbone. Density functional theory (DFT) calculations have been carried out for Mulliken atomic charge information and hydrogen atom free energy of adsorption (ΔG_H_) in each polymer. As shown in Figure 5f, the sulfonyl groups in FSO perfectly act as electron‐output “tentacles”, which help to export electrons to the proton or co‐catalyst to drive the photocatalytic reaction. This work focuses on the electron export pathways in photocatalytic processes to support the understanding of polymer photocatalytic active sites. In addition, Zhang et al. have explored the influence of different substituents on the photocatalytic performance of D‐A polymers employing FSO as an acceptor nucleus. The introduction of F atoms with strong electron‐absorbing ability on the acceptor core can further promote the electron acceptability of FSO, leading to an effective separation of photogenerated excitons.^[^ ^79^ ^]^

To understand the effects of monomer composition, chemical structure, and other details on photocatalytic behavior, Zhang and colleagues have inserted a benzene “π‐bridge” between the FSO and Py units.^[^ ^75^ ^]^ A series of D‐π‐A CMPs photocatalysts with different chemical compositions have been synthesized utilizing the statistical copolymerization scheme. Statistical copolymerization methods for optimizing the polymer structure and composition ratios to enhance the photocatalytic activity of D‐A polymers have been confirmed by this work. As in Figure 5g, a broader spectrum responsive polymer has been further constructed by replacing the benzene “π‐bridge” with a narrow band gap thiophene “π‐bridge”. Three pairs of D‐π‐A polymers with different donor and acceptor units (all containing thiophene blocks) have been fabricated successfully, proving the generalizability of this strategy.

#### 2.2.2 Based on Triazine

**Figure 6:**
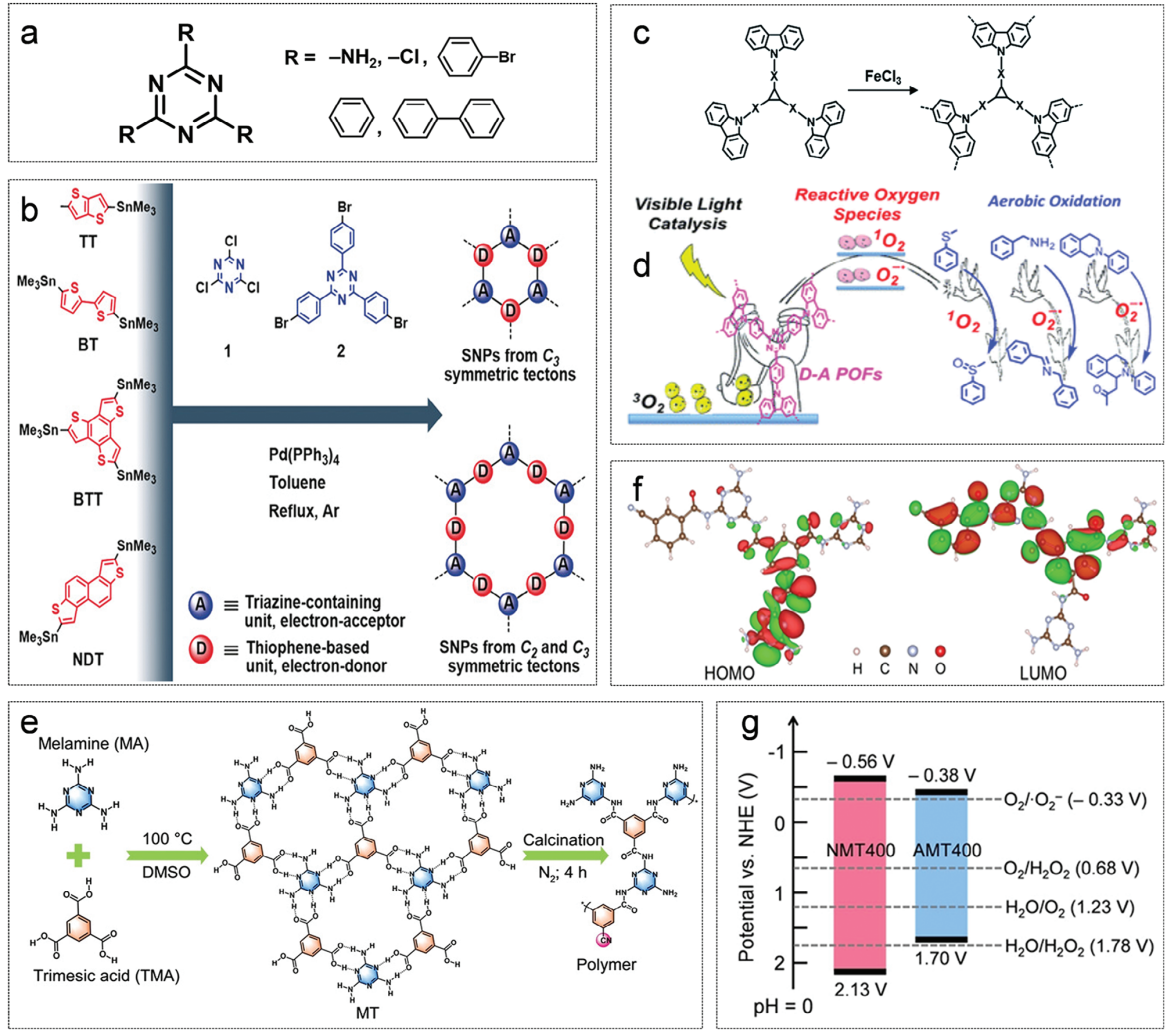
D‐A conjugated polymers based on triazin acceptor. a) The chemical structure of triazine and its derivatives. b) Synthetic pathway toward sulfur and nitrogen‐containing porous polymers. Reproduced with permission.^[^ ^80^ ^]^ Copyright 2018, Wiley‐VCH. c) Chemical structure of carbazole‐triazine based CMPs. d) The mechanism of active oxygen generation. Reproduced with permission.^[^ ^81^ ^]^ Copyright 2018, Royal Society of Chemistry. e) The schematic for the preparation of D‐A polymers. f) The distribution of HOMO and LUMO wave functions of NMT400. g) Band gap of AMT400 and NMT400. Reproduced with permission.^[^ ^82^ ^]^ Copyright 2022, Wiley‐VCH.

Triazine molecules (**Figure** **6**a), acting as indispensable nodes in a 2D or 3D framework, have attracted increasing attention and have been widely used in studies of photocatalytic hydrogen evolution and CO_2_ reduction. Eight CMPs based on thiophene derivatives (D) and triazine derivatives (A) have been designed by Michael J. Bojdys' group.^[^ ^80^ ^]^ As shown in Figure 6b, they show a similar pore structure and exhibit a maximum photocatalytic hydrogen evolution rate of 3158 µmol g^−1^ h^−1^ and an apparent quantum yield of 4.5%. The strong and weak charge transfer (CT) effects are achieved by modulating the presence or absence of the spacer building block (benzene) between D‐A and breaking the commonly accepted misconception that strong CT interactions are harmful in bandgap engineering. Zhang et al. have reported a series of carbazole‐triazine‐based D‐A CMPs where a stable CT state has formed between the D‐A fragments in the CMPs (Figure 6c) and enabled an efficient triplet‐triplet energy transfer to generate ^1^O_2_.^[^ ^81^ ^]^ Moreover, the variation of the D‐A distance leads to a tunable redox potential in the ground and excited states, resulting in the regulation of reactive oxygen species (ROS) production (Figure 6d). As exemplified in Figure 6e, cyanide‐based porous nanorod‐like polymers are obtained by calcining supramolecular assemblies of melamine (MA) and trimesic acid (TMA) under air.^[^ ^82^ ^]^ The calcination atmosphere has been applied to modulate the electronic energy band structure (Figure 6f,g), allowing for simultaneous two‐electron water oxidation and two‐electron oxygen reduction reactions. DFT calculations also demonstrate that the adsorption and activation of O_2_ on cyanide groups are driven more readily in the microstructurally adjusted polymer.

#### 2.2.3 Based on Benzothiadiazole (BT)

**Figure 7:**
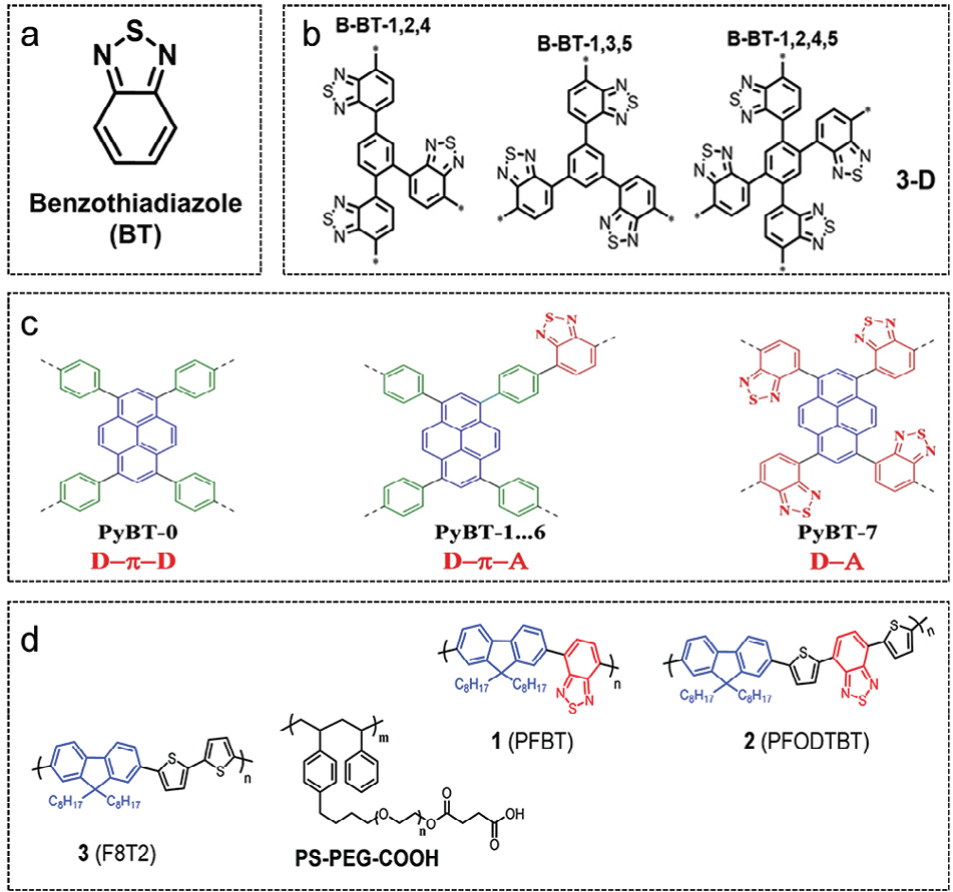
D‐A conjugated polymers based on BT acceptor. a) The chemical structure of the BT molecule. b) Structures of polybenzothiadiazoles with different molecular designs. Reproduced with permission.^[^ ^83^ ^]^ Copyright 2016, Wiley‐VCH. c) The polymer molecules with D‐A and D‐π‐A structures. Reproduced with permission.^[^ ^84^ ^]^ Copyright 2018, Elsevier. d) Chemical structure of polymer dots. Reproduced with permission.^[^ ^85^ ^]^ Copyright 2017, Royal Society of Chemistry.

Benzothiadiazole (BT) as a classical acceptor can be coupled with various conjugation units to build extended π‐conjugation systems, the structure of BT is shown in **Figure** **7**a. As shown in Figure 7b, a series of polymers with 1D linear or 3D network structures have been synthesized by Wang et al. by altering the substitution position of the BT unit on the benzene ring.^[^ ^83^ ^]^ Jiang's group has reported an aggregate library of D‐π‐A CMPs photocatalysts with Py, BT, and benzene (biphenyl) as D, A, and π cross‐linking units, respectively.^[^ ^84^ ^]^ The polymer structure in Figure 7c. Differences in hydrogen evolution performance have revealed that the type of π‐crosslinker, the D‐π‐A molecular structure, and the D‐A ratio play an essential role in the photocatalytic process. Tian et al. show that the interactions between different BT units in the polymer sites cause the formation of hydrogen to be kinetically more favorable.^[^ ^85^ ^]^ As shown in Figure 7d, the N‐sites in the BT units play a crucial activation role in the formation of hydrogen molecules.

The construction of D‐A polymers or conjugated microporous polymers through molecular strategies such as side‐chain engineering and chemical substitution has been widely demonstrated to be a reliable approach to facilitate charge separation and transport. However, the current number of acceptor molecules is still too small, which limits the exploration of high‐performance D‐A polymers, and for this reason, ongoing development of novel electron acceptor materials is needed. Secondly, polymers with tunable photoactivity lack the structure for long‐range ordering.^[^ ^86^ ^]^ The disordered structure might limit the transport of excited state electrons to its surface, and on the other hand, it is difficult to help researchers construct the relationship between the photocatalyst and its catalytic activity at the molecular level. In addition, it is undeniable that there are several differences in the photocatalytic performance of polymers with different physical properties (such as molecular weight, degree of branching, and termination), and the controlled preparation of polymers is necessary.^[^ ^45^, ^87^ ^]^

### 2.3 D‐A Type Crystalline Polymers

#### 2.3.1 Covalent Organic Frameworks (COFs)

Covalent organic frameworks (COFs) are defined as porous organic crystalline materials linked by covalent bonds with a designable topology, tunable pore size, and abundant active sites.^[^ ^88^ ^]^ These frameworks predominantly consist of rigid π‐conjugated units, facilitating the formation of topologically ordered π arrays, triggering electronic coupling in the conjugate direction as well as promoting charge carrier transport. However, photogenerated e^−^ and h^+^ in native COFs make it easier to undergo undesired recombination during transport, thereby adversely affecting their application in photocatalysis.^[^ ^89^ ^]^ To address this limitation, the D and A units are introduced into the backbone for improving the e^−^ and h^+^ transport pathways and expanding the optical absorption of COFs. In recent years, high success has been achieved through the D‐A COFs strategy, as it combines the exceptional photocatalytic performance of polymers with the distinct advantages offered by COFs, including high crystallinity and specific surface area.^[^ ^90^ ^]^ In this section, the most representative imine‐linked and fully π‐conjugated COFs containing D‐A building blocks and their progress in photocatalysis are reviewed.

##### Imine‐Linked D‐A Type COFs

The imine‐linked COFs with good chemical stability are synthesized from aldehydes and aromatic amines with the participation of Lewis acids or organic acids. Normally, most of the imine‐linked COFs are capable of maintaining open pores under common organic solvents, water, and various acid and base conditions. Meanwhile, the imine bond‐linked COFs possess good crystallinity and simple preparation conditions.^[^ ^91^ ^]^ Well‐structured stability and easy batch preparation properties allow imine‐linked D‐A type COFs to be most widely investigated in the photocatalysis field.

**Figure 8:**
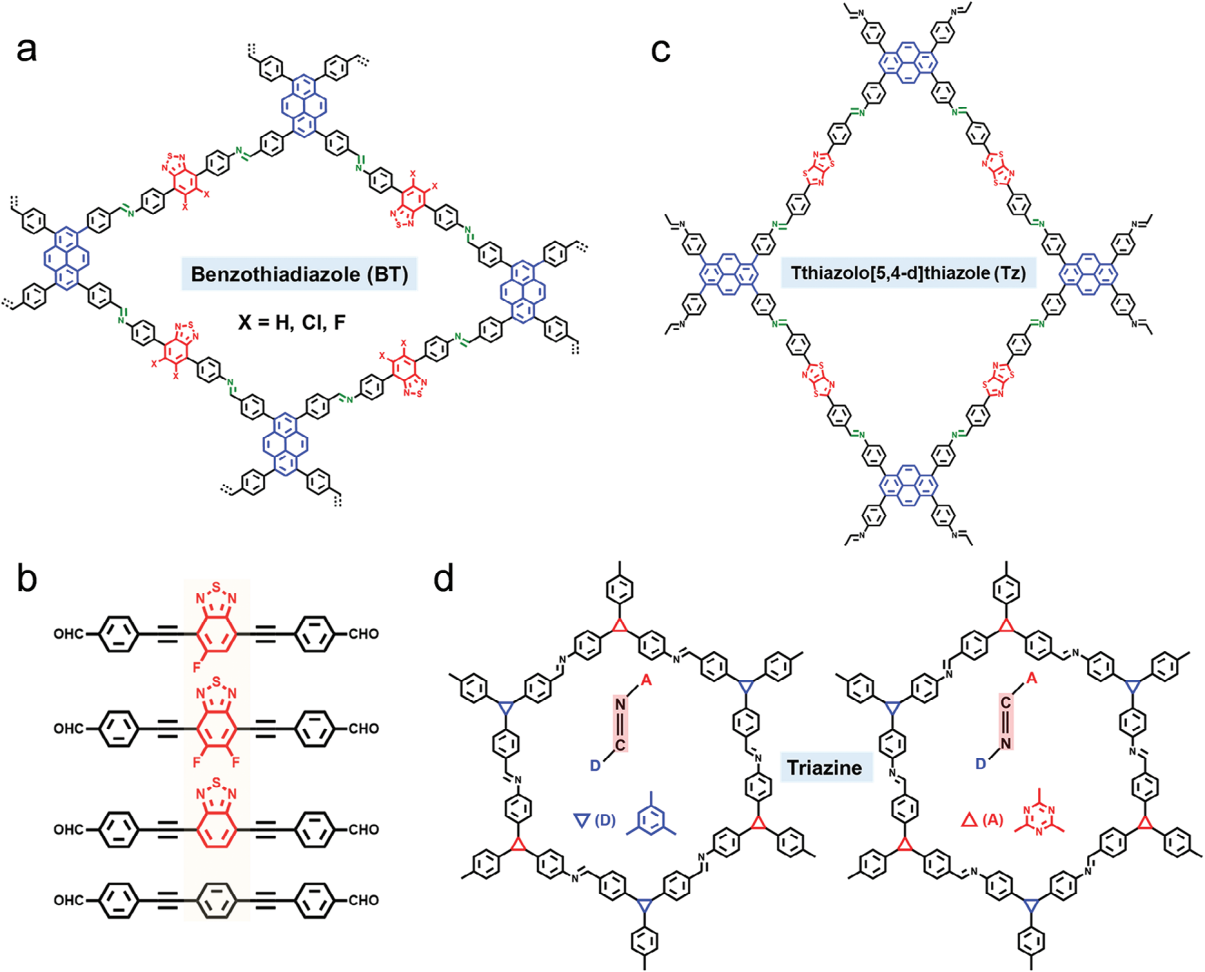
Imine‐linked D‐A type COFs with different structures. a) Bonding structure of Py‐XTP‐BT‐COFs based on BT acceptor. b) Monomers of the NKCOFs. c) Bonding structure of PyTz‐COFs based on Tz acceptor. d) The chemical backbones of the isomeric COFs based on triazine acceptor.

Based on benzothiadiazole (BT): Structural fine‐tuning of photocatalysis‐active Py‐HTP‐BT‐COFs (the structure is shown in **Figure** **8**a) by Chen's group through fluorination and chlorination strategies have yielded a significant increase in the rate of photocatalytic hydrogen evolution.^[^ ^92^ ^]^ Theoretical calculations have further demonstrated that charge recombination is effectively suppressed via halogenation of the acceptor (BT) unit and the activation energy for the formation of intermediate species (H*) on the surface of COFs has been significantly reduced. Inspired by photosystem I, NKCOF photocatalysts based on Schiff‐base reaction have been designed by Zhang et al. The highly ordered 2D structure reveals enough active sites are available for hydrogen evolution.^[^ ^93^ ^]^ As shown in Figure 8b, the alkyne groups are introduced into the COFs as connecting bridges between D‐A to facilitate the photogenerated carrier separation. The imine‐linked D‐A‐D structure of COFs has been prepared by Dong et al. by using BT as the acceptor unit, and the rate of photocatalytic hydrogen evolution under visible light (AM 1.5G) is 5458 µmol g^−1^ h^−1^.^[^ ^94^ ^]^

Based on thethiazolo[5,4‐*d*]thiazole (Tz): Compared with the BT acceptor, the stronger ability of electron‐pulling and the coplanar geometry of thethiazolo[5,4‐*d*]thiazole (Tz) molecule allow for the construction of highly active D‐A type COFs photocatalysts. Tz binding to the backbone of COFs is an attractive strategy to extend the light absorption range. Lotsch et al. compare the UV–vis absorption spectra of Tz‐containing units with COFs without Tz, and TpDTz‐COFs have a red‐shifted absorption band edge up to 600 nm compared to TpDTp‐COFs.^[^ ^95^ ^]^ As shown in Figure 8c, a new D‐A structure of COFs (PyTz‐COFs) has been reported by Yang and his co‐workers, constructed from Tz and Py. PyTz‐COFs show high photoelectric activity in photocatalytic (arylmethyl)amines coupling and persistent activity in sunlight‐driven photocatalytic hydrogen evolution.^[^ ^96^ ^]^

Based on triazine: Triazine units have been found to not only stabilize the negative charges generated in COFs after photoexcitation but also to form D‐A interactions to improve the charge separation efficiency.^[^ ^97^ ^]^ Meanwhile, the role of triazine as a photocatalytic active center has been demonstrated by the study of Cai et al.^[^ ^98^ ^]^ From the perspective of constitutional isomerism of the linkages, D‐A COFs with different imine bond orientations are synthesized by Arne Thomas et al.^[^ ^99^ ^]^ As in Figure 8d, for one type, the donor connects with imine carbon, and for the other type, connects with imine nitrogen. The constituent isomers exhibit significant differences in photophysical properties as well as photocatalytic performance. The effect of the constitutional isomerism in COFs on photocatalytic performance proposed in that study is very general and should be considered during the optimization and design of the photophysical‐chemical properties of COFs. The exciton binding energy (*E*_b_) of ten imine‐linked D‐A COFs formed by the condensation of amine and aldehyde monomers have been calculated by Jiang et al. using DFT theory,^[^ ^100^ ^]^ and *E*_b_ presents distinctly different variation trends. Four COFs with similar structures and energy levels, TAPB‐OMe‐COF, TAPT‐Cl‐COF, TAPT‐H‐COF, and TAPT‐OMe‐COF have been synthesized experimentally under the guidance of such theory, and the difference in their photocatalytic hydrogen evolution activity is consistent with the *E*_b_ predicted. This research work has provided a paradigm for the design and synthesis of targeted COFs.

##### Full π‐Conjugated D‐A Type COFs

As mentioned above, imine‐linked COFs are widely used for photocatalysis due to their excellent crystallinity and abundant monomeric molecular resources. Owing to the local conjugation property of imine bonds, imine‐linked COFs cannot possess obvious advantages in carrier transport.^[^ ^101^ ^]^ Therefore, it might be an effective path to enhance photocatalytic performance through constructing COFs with better photoelectric performance.

**Figure 9:**
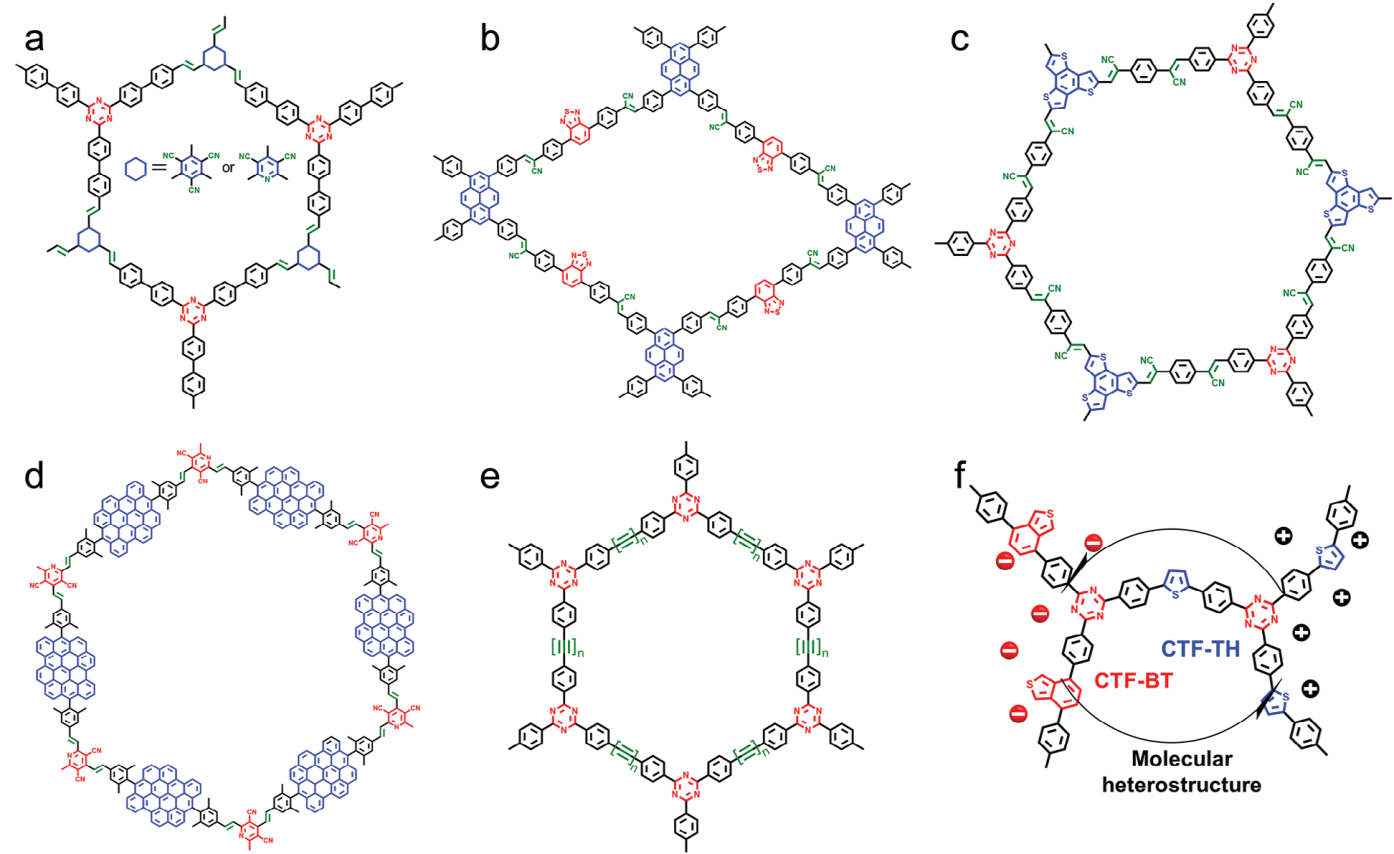
Full π‐conjugated D‐A type COFs with different structures. a) The structure with different geometrical symmetries based on vinylene‐linked COF. b) The structure of the Py‐BSZ‐COF. c) The structure of COF‐JLU35. d) The topology‐directed synthesis of the BDOV‐COFs with vinylene‐linked. e) The structure of triazine‐linked D‐A type COFs containing acetylene. f) The D‐A structured CTFs by sequential polymerization.

Vinylene‐linked D‐A type COFs: Vinylene‐linked COFs’ backbone composed of fully conjugated carbon–carbon double bonds avoids the distortion of spatial site barrier caused by single bond linkage and contributes to a better plane π‐conjugated structure. In general, vinylene‐linked COFs are generated by Aldol condensation reactions or Knoevenagel condensation reactions. As shown in **Figure** **9**a, the photophysical properties of COFs are regulated in terms of the geometric symmetry of the building block molecules by Zhang et al. Their group form octupolar characters vinylidene‐linked g‐C_54_N_6_‐COF employing D_3h_‐symmetric monomer molecules via Knoevenagel condensation.^[^ ^102^ ^]^ The g‐C_54_N_6_‐COF shows better photoinduced charge generation, migration, and separation than the geometric symmetry deficient g‐C_52_N_6_‐COF, with excellent photocatalytic oxygen and hydrogen evolution capabilities. Li et al. have constructed the fully conjugated Py‐BSZ‐COF with BT and Py as the construction blocks (Figure 9b).^[^ ^103^ ^]^ Superoxide radicals (·O_2_^−^) are efficiently produced in Py‐BSZ‐COF under visible light irradiation for the photocatalytic oxidative amine coupling and cyclization of thioamide to 1,2,4‐thiadiazole. The three component D‐π‐A structure has been developed by Liu et al. through the Knoevenagel condensation and Schiff base condensation to bond ethylene and imine bonds into the COFs’ backbone, respectively. As shown in Figure 9c, triazine and benzotrithiophene as D and A units. COF‐JLU35 demonstrates a photocatalytic hydrogen evolution rate of up to 70.8 mmol g^−1^ h^−1^,^[^ ^104^ ^]^ which is one of the highest‐performance COF photocatalysts to date.

In 2019, unsubstituted vinylene‐linked COFs were synthesized by Zhang et al. using an aldol condensation reaction. Afterward, the BDOV‐COFs (dibenzo[*hi*, *st*]ovalene) with vinylene‐linked have been prepared by Hai I. Wang and co‐workers.^[^ ^105^ ^]^ Controllable band gap nanographene (Figure 9d) is introduced into the main chain by aldol condensation, and a unique COF structure with ABC stacking is obtained. The exposure of DBOV cores in COF pores enables the guest molecules to combine effectively with the reaction sites and show significant photocatalytic activity in the hydroxylation.

Triazine‐linked D‐A type COFs: Triazine COFs are usually referred to as crystalline covalent triazine frameworks (CTFs) with the main chains connected by –C = N bonds, which are also typical of all‐π conjugated COFs. The CTFs have high chemical stability and a robust pore structure because of the absence of weak bonds in the backbone. In addition, the construction of D‐A type COFs can be simplified by using triazine molecules as acceptor units. As shown in Figure 9e, a couple of CTFs containing acetylene, namely CTF‐EDDBN and CTF‐BDDEN,^[^ ^106^ ^]^ are synthesized by Xu et al. The acetylene and diacetylene portions of the CTFs are essential to modulate the energy band structure and restrain e^−^‐h^+^ combining. At the same time, a significant decrease in the Gibbs free energy (ΔG) of intermediate species (OH*) formation has been achieved in the presence of alkyne groups. The final realization of the efficient production of H_2_O_2_ through a two‐electron water oxidation pathway and two‐electron oxygen reduction pathway. The studies of Zhang et al. further demonstrate the vital role of acetylene building blocks in the photocatalytic process. The acetylene part in extended D‐π‐A conjugation minimizes exciton binding energy, promotes exciton delocalization, and enhances carrier lifetime, which facilitates the positive charge transfer and separation.^[^ ^107^ ^]^ Li et al. have obtained D‐A structured CTFs by sequential polymerization using BT and thiophene as D and A units,^[^ ^108^ ^]^ the molecular heterogeneous structure as shown in Figure 9f exhibits a significantly higher photogenerated carrier separation efficiency.

#### 2.3.2 Metal‐Organic Frameworks (MOFs)

Metal‐organic frameworks (MOFs), as a type of crystalline porous coordination materials, have gained extensive attention in various fields, including gas separation, drug delivery, sensing, and heterogeneous catalysis.^[^ ^112^ ^]^ This is primarily due to their remarkable features, such as large surface areas, tunable geometric shapes, exposed active sites, and versatile post‐modification capabilities. Additionally, MOFs exhibit unique exciton dissociation and charge transfer mechanisms, alongside their highly ordered crystalline structure, making them attractive candidates for potential applications in the field of photocatalysis.^[^ ^113^ ^]^ The significant interest in MOFs' photocatalytic properties is evident from the annual publication of numerous research and review papers.^[^ ^114^, ^115^, ^116^ ^]^ However, incorporating both electron acceptors and donors as ligands within the same MOF for photocatalysis presents a notable synthetic challenge.

**Figure 10:**
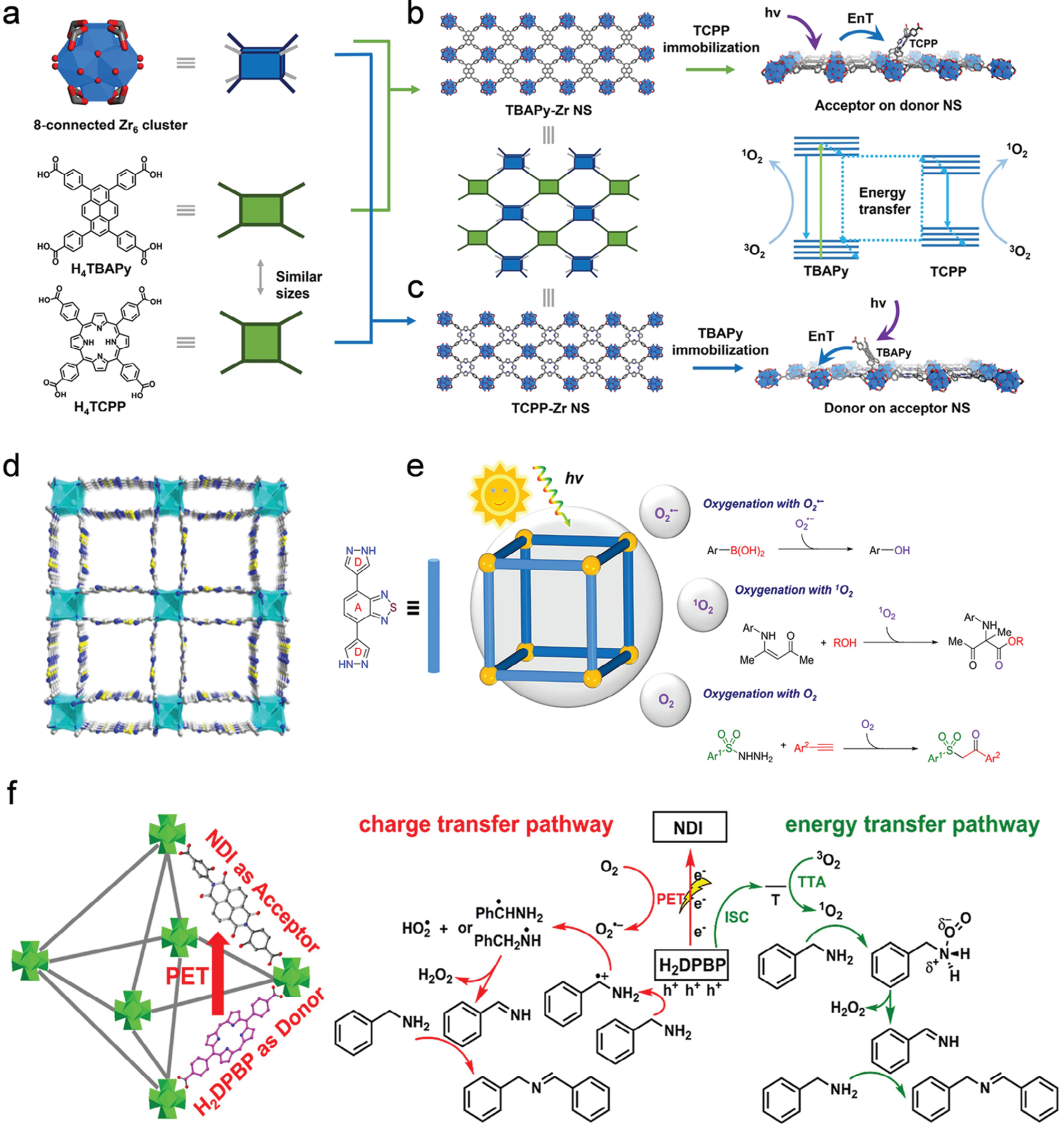
The D‐A type MOFs with different structures. a) Schematic diagrams for the construction of MOFs NS. b) Schematic diagram of the acceptor‐on‐donor NS. c) Schematic diagram of the donor‐on‐acceptor NS. Reproduced with permission.^[^ ^109^ ^]^ Copyright 2021, Wiley‐VCH. d) Crystal structure of JNU‐204. e) Schematic illustration of JNU‐204 as photocatalyst for aerobic oxygenation. Reproduced with permission.^[^ ^110^ ^]^ Copyright 2021, American Chemical Society. f) The structure and proposed photocatalytic reaction mechanism of D‐A type MOF Zr‐NDI‐H_2_DPBP. Reproduced with permission.^[^ ^111^ ^]^ Copyright 2023, American Chemical Society.

As shown in **Figure** **10**a, Pan et al. have prepared two MOF nanosheets with excellent charge separation properties, namely donor‐on‐acceptor NS (Figure 10c) and acceptor‐on‐donor NS (Figure 10b) catalysts, using tetrakis(4‐carboxyphenyl) porphyrin (H_4_TCPP) and 1,3,6,8‐tetrakis(p‐benzoic acid)pyrene (H_4_TBAPy) as acceptor and donor, respectively.^[^ ^109^ ^]^ As the results show, the second mode exhibits a higher catalytic performance than donor‐on‐acceptor NS due to the faster initial reaction kinetics induced by site‐isolated active centers. Although this is not a D‐A model in the strict sense, this work still demonstrates the importance of D‐A interactions for photocatalytic reactions in terms of energy transfer. Lu et al. have reported microporous wuk‐topology Zn‐MOF (JNU‐204, Figure 10d) with the D‐A type photosensitizer pyrazole‐benzothiadiazole‐pyrazole as a ligand,^[^ ^110^ ^]^ which is a molecule with a D‐A‐D conjugated system for rapid separation of photocatalytic excitons. As shown in Figure 10e, JNU‐204 has achieved photocatalysis hydroxylation of 4‐cyanophenylboronic acid and hydroxylation of arylboronic acid with high yields. β‐ketosulfone is present in the structural backbone of biologically active molecules, and the synthetic methods developed to date have been primarily through homogeneous catalysis. The β‐ketosulfone is obtained by a non‐homogeneous process with moderate to good yields (60‐80%) when JNU‐204 is utilized as the photocatalyst. Pyrrolo[2,1‐*a*]isoquinoline‐containing heterocycles have been further successfully synthesized through a series of photocatalytic oxidation processes, confirming that the JNU‐204 is an effective non‐homogeneous photocatalyst.

Gu et al. have constructed a D‐A type ligand MOF (Zr‐NDI‐H_2_DPBP) by embedding the donor 5,15‐di(*p*‐benzoato)porphyrin (H_2_DPBP) into a naphthalene diimide (A)‐based Zr‐MOF via a one‐step method (Figure 10f).^[^ ^111^ ^]^ Zr‐NDI‐H_2_DPBP displays an imine generation rate (136 mmol g^−1^ h^−1^) that is far superior to that of other noble metal‐free MOF photocatalysts when it is used as a non‐homogeneous photocatalyst for the benzylamine oxidative coupling reaction. Combining theoretical calculations and experimental results, the enhanced photocatalytic performance is attributed to the photoelectron transfer from H_2_DPBP to NDI and the energy transfer generated by the donor H_2_DPBP. Energy transfer and charge transfer produce abundant singlet oxygen (^1^O_2_) and superoxide radicals (·O_2_^−^) while improving the efficiency of e^−^‐h^+^ separation. This work offers a pathway for D‐A type MOF materials to be applied to facilitate solar energy conversion.

The synthesis of MOFs either by introducing a D‐A molecule as a single ligand into the framework or by taking a donor and acceptor molecule as a dual ligand is a difficult challenge, as it involves several issues such as crystallinity, D‐A coordination, degree of conjugation, and solubility simultaneously. Therefore, there are fewer literature reports about D‐A type photocatalytic MOFs.

#### 2.3.3 Post‐Modified Crystalline Polymers

The open pores of the framework material are suitable for the encapsulation of guest molecules, metal clusters, and single atoms. As an alternate way, the formation of D‐A interactions by limiting the electron donor/acceptor spatially within the open channels of a crystalline framework material is an effective method for preparing D‐A materials. Also, the dispersion and domain limitation of metal clusters or single atoms in the D‐A type framework materials may enhance photocatalytic activity and selectivity.

**Figure 11:**
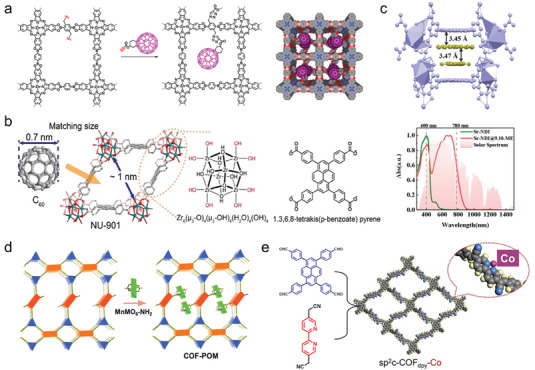
Post‐modified strategy to construct D‐A type crystalline polymers. a) Schematic of converting open lattice into segregated D‐A COF. Reproduced with permission.^[^ ^117^ ^]^ Copyright 2014, American Chemical Society. b) Immobilization of C_60_ within the pores of NU‐901. Reproduced with permission.^[^ ^118^ ^]^ Copyright 2023, Wiley‐VCH. c) Structure and absorption spectra of D‐A type MOF cocrystal. Reproduced with permission.^[^ ^119^ ^]^ Copyright 2023, Wiley‐VCH. d) Schematic representation of POM clusters by covalent binding in D‐A type COF. Reproduced with permission.^[^ ^120^ ^]^ Copyright 2022, American Chemical Society. e) The structure of monatomic Co‐modified D‐A type COFs, sp^2^c‐COF_dpy_‐Co. Reproduced with permission.^[^ ^121^ ^]^ Copyright 2020, Elsevier.

As shown in **Figure** **11**a, Jiang et al. have reported a synthetic strategy to convert an open framework structure into an ordered D‐A structure, that is, the acceptor molecules, fullerenes, are anchored within the nanochannels of COFs by covalent bonding.^[^ ^117^ ^]^ The D‐A system triggers light‐induced electron transfer, while time‐resolved electron spin resonance spectroscopy (TR‐ESR) confirms the fullerene content is essential for charge separation. This work has demonstrated the viability of the post‐modified strategy to construct D‐A framework materials and its potential advantages in photocatalytic charge separation. In general, MOF materials with more defined crystal structures and reactant transport channels have achieved remarkable success in photocatalysis. As mentioned in Section 2.3.2, direct construction of D‐A type photocatalytic MOFs is less reported, instead, the post‐modified strategy is an ideal way to construct D‐A type MOFs. As shown in Figure 11b, the C_60_ is encapsulated into a Zr‐based MOF (NU‐901) by Meng et al.^[^ ^118^ ^]^ A powerful built‐in electric field has been induced by the inhomogeneous charge distribution in C_60_@NU‐901, and the photocatalytic hydrogen evolution rate reaches 22.3 mmol g^−1^ h^−1^, which is one of the highest values for MOFs. At the same time, it facilitates charge transport along the C_60_ and MOF channels, which accelerates charge‐transport kinetics. Unfortunately, C_60_@NU‐901 does not form homogeneous crystals, due to the intrinsic limitations of MOFs.

A suitable framework is essential for the growth of D‐A type MOF crystals, and it will also greatly help us to understand the elements of charge separation, transport, and reactive active sites in photocatalysis. Duan et al. have constructed D‐A type MOF crystals by encapsulating the electron donor anthraquinone (AQ) into the ligand channel of NDI‐based MOF (Sr‐NDI) using a host‐guest strategy.^[^ ^122^ ^]^ Compared to AQ and Sr‐NDI, Sr‐NDI@AQ shows better yields in the coupling reaction between aldehyde and phenyl vinyl sulfone. As shown in Figure 11c, Zhang et al. have formed unique MOF cocrystal materials by inserting donor molecules into the ligands of MOFs at the molecular level.^[^ ^119^ ^]^ The strong CT interactions not only broaden the absorption spectrum into the infrared optical region, but also shorten the intermolecular stacking distance, allowing the photogenerated carriers to be smoothly transported in the guest molecules, frameworks, and coordination metals, and realizing the photocatalytic performance from scratch. This post‐modified strategy for building D‐A type framework materials is effective, but its stability is something that needs to be further improved.

The metal clusters or single atoms are encapsulated into the nanopores is an exciting method to further enhance the photocatalytic performance of D‐A crystalline porous materials, and has become an effective tool to study the relationship between structure and catalytic performance, and has been widely investigated. A fluorinated COFs (TAPT‐TFPA‐COFs), where electronegative fluorine strongly confines Pd‐isolated clusters (Pd‐ICs) by enhancing metal‐support interactions, has been reported by Guo et al.^[^ ^123^ ^]^ TAPT‐TFPA COFs@Pd ICs kept a good H_2_O_2_ generation rate (2143 µmol h^−1^ g^−1^) during the photocatalytic process of up to 100 h, and the content of Pd‐ICs remained stable without almost any metal leaching. In comparison with supramolecular interaction forces such as van der Waals force, hydrogen bonding, and Coulomb force, metal clusters stabilized in pores by covalent bonding are undoubtedly the most stable. As shown in Figure 11d, Lan et al. have reported the homogeneous dispersion of polyoxometalates (POMs) into D‐A type COFs using Schiff base reaction.^[^ ^120^ ^]^ In photocatalytic CO_2_ reduction, the D‐A type TTF‐TAPT COF acts as CO_2_ enrichment and light absorption, and the MnMo_6_ cluster functions as a reaction site. Specifically, photogenerated electrons are transferred to MnMo_6_ clusters with CO_2_ reduction activity via covalent bonds, and holes are enriched in TTF‐TAPT COFs, for CO_2_ reduction and H_2_O oxidation, respectively. This work has achieved highly active photocatalytic CO_2_ reduction and H_2_O oxidation by covalently bonded POM and COF by combining light absorption, electron transfer, and active sites. Wang et al. have synthesized sp^2^c‐COF_dpy_ (Figure 11e), a D‐A type COFs material with excellent extended aromatic conjugation and redox active sites, through the Knoevenagel polycondensation reaction.^[^ ^121^ ^]^ The monoatomic Co is further anchored in embedded bipyridine units to provide catalytically active sites for CO_2_ photo‐reduction. As optimized, sp^2^c‐COF_dpy_‐Co with monoatomic Co sites perform 0.99 mmol h^−1^ g^−1^ conversion and 81.4% high selectivity in the reduction of CO_2_ to CO under the condition of no co‐catalysts and photosensitizers. This work has demonstrated the cascade effect of oriented electron delivery and intramolecular electron delocalization due to nanostructures in D‐A type COFs, providing a reduced‐oxidation basis for CO_2_ conversion.

In comparison to normal polymers, the local crystallinity of crystalline polymers plays an important role in carrier transport and slowing down e^−^‐h^+^ recombination. MOFs with high crystallinities, micro‐ and nanochannels, and metal sites have natural advantages in photocatalysis. However, the direct construction of D‐A type MOFs is difficult and requires more effort from researchers. The COFs have a larger pore size and a substantially larger contact area with various reaction substrates. However, most COFs require the introduction of metal‐active sites in ligands or pores, or the addition of sacrificial agents to the system to enhance their photocatalytic performance, something that is not in line with the ideal concept of green sustainability.^[^ ^124^ ^]^ Furthermore, the D‐A type COFs constructed based on different covalent bond types can exhibit efficient photocatalytic performance, and the effect of the bonding mode (imine, boronic ester, vinylene, and so on) on the catalytic performance in the D‐A type COFs has not been fully explained.^[^ ^125^ ^]^ Moreover, making single crystals of D‐A type COFs by further optimizing the growth method is significant for revealing the photocatalytic mechanism.^[^ ^89^ ^]^

The encapsulation of organic molecules into pores to construct D‐A systems is a powerful tool for enhancing the photocatalytic performance of pristine porous materials, which is inextricably linked to the charge separation effect and the synergistic effect between the host and guest. Similarly, the encapsulation of metal clusters or single atoms can take full advantage of their well‐defined electronic and geometrical structures to provide more reactive active sites for redox and to compensate for the low selectivity of organic materials. However, it should be noted that the D‐A system based on noncovalent bonding is prone to partial dissolution during photocatalysis, leading to crystal structure destruction.^[^ ^126^ ^]^ Therefore, improving the photostability is a problem to be addressed.

## 3 Intermolecular D‐A Interactions

In the field of intermolecular interactions, a major challenge is related to the typical non‐covalent interaction forces, including π‐interactions (σ–π, C‐H···π, π–π stacking), van der Waals forces (induction and dispersion force), electrostatic forces, hydrogen bonds, and halogen bonds. Despite their indispensable roles in shaping the structure, functionality, and stability of organic materials, the precise prediction and quantification of these interactions remain elusive.^[^ ^127^ ^]^ To address this issue, researchers have turned to supramolecular and heterojunction engineering as effective strategies for manipulating organic photocatalysts, enabling the indirect capture and analysis of extrinsic photocatalytic properties that shed light on intermolecular interactions. In addition, these approaches offer opportunities for widening the absorption spectra, optimizing exciton dynamics, and minimizing losses arising from non‐radiative pathways.^[^ ^38^ ^]^ In this context, we focus on the ongoing research progress involving supramolecular and D‐A polymer heterojunction strategies in photocatalysis.

### 3.1 Two‐Component Supramolecules

**Figure 12:**
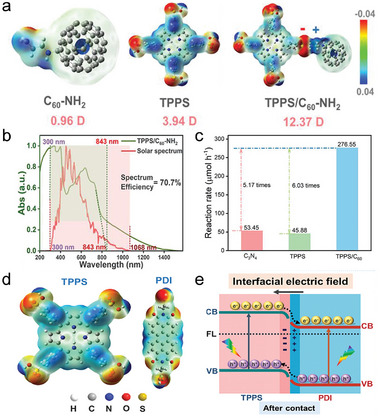
The D‐A type two‐component supramolecules with different structures. a) The electrostatic potential distribution and molecular formula of TPPS, C_60_‐NH_2_, and TPPS/C_60_‐NH_2_. b) The UV–vis absorption spectroscopy of TPPS/C_60_‐NH_2_. c) The comparison of the hydrogen evolution rates of different materials. Reproduced with permission.^[^ ^127^ ^]^ Copyright 2021, Wiley‐VCH. d) The electrostatic potential distribution and molecular formula of PDI and TPPS. e) Scheme of charge transport route at the D‐A interface in TPPS/PDI. Reproduced with permission.^[^ ^128^ ^]^ Copyright 2022, Wiley‐VCH.

The large dipole moment and high molar extinction coefficient of the molecular porphyrins enable them to obtain excellent absorption of visible light. In addition, porphyrins possess π‐conjugated chromophores, rigid planes, and easily modifiable molecular structures. Porphyrins have always been considered a versatile platform for building various types of optoelectronic materials due to these natural advantages. As illustrated in **Figure** **12**a, Zhu's group has constructed the D‐A supramolecular material TPPS/C_60_‐NH_2_ taking porphyrin as the D unit and the C_60_ with a high degree of π‐electron delocalization on the 3D surface as the A unit.^[^ ^127^ ^]^ As in Figure 12b, the full spectrum of visible light (300–850 nm) absorption has been successfully achieved in TPPS/C_60_‐NH_2_ with a theoretical spectral efficiency of up to 70%. Meanwhile, the large IEF created between the D‐A molecules accelerates the charge separation significantly and results in a long lifetime charge separation state. The final photocatalytic hydrogen evolution rate of TPPS/C_60_‐NH_2_ is as high as 34.57 mmol g^−1^ h^−1^, which surpasses that of most organic photocatalytic materials (Figure 12c). Taking advantage of the huge tunability of the functional groups and electronic structures of the two organic molecules, ZnTCPP/C_60_‐EDA, a D‐A supramolecular material obtained by linking tetrakis (4‐carboxyphenyl) zinc porphyrin (ZnTCPP) with ethylenediamine functionalized C_60_ (C_60_‐EDA) through electrostatic interactions, has been established successfully.^[^ ^129^ ^]^ Conversion of the ZnTCPP to C_60_‐EDA electron transfer channel has been effectively established, leading to a significant improvement in the separation of photogenerated excitons and exhibiting a photocatalytic hydrogen evolution performance of 16.2 mmol g^−1^ h^−1^.

With the advantages of strong electron affinity, wide spectral absorption range, and stable chemical structure, perylene diimide and its derivatives (PDI) are widely applied in the fields of photocatalysis, photothermal conversion, and organic solar cells. The D‐A photocatalyst TPPS/PDI with high charge separation efficiency is constructed by Zhu et al. by choosing PDI as the electron acceptor and tetra(4‐sulfonatophenyl)porphyrin (TPPS) as the donor (Figure 12d).^[^ ^128^ ^]^ As illustrated in Figure 12e, the long lifetime of the excited state has been induced successfully by the formation of the D‐A interface, which drives the photogenerated electrons to participate in reduction reactions. The photocatalytic hydrogen evolution rate of TPPS/PDI is up to 30.36 mmol g^−1^ h^−1^, being 9.41 and 9.95 times higher than that of pure PDI and TPPS, respectively.

### 3.2 Molecular Cocrystal

**Figure 13:**
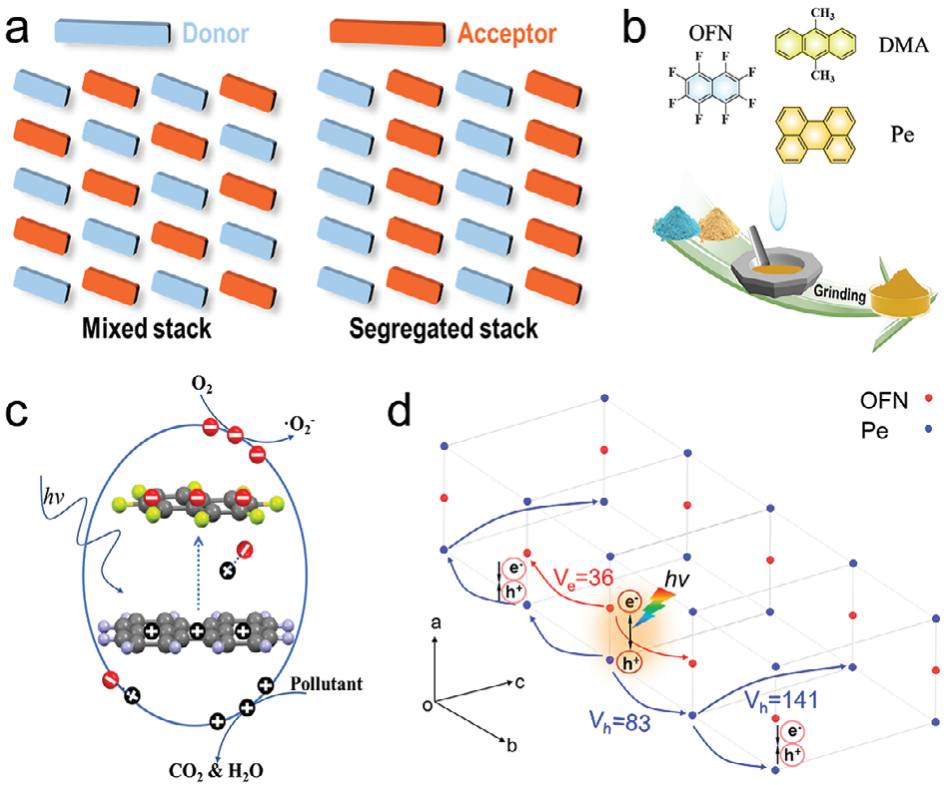
The D‐A type molecular cocrystal for photocatalysis. a) The common molecular stacking patterns in molecular cocrystals: Mixed or segregated stack. b) Diagram of large‐scale preparation and structure of molecular cocrystal. c) Schematic of the photocatalytic process in molecular cocrystal. d) Electron and hole transfer pathway based on the calculation of electronic coupling in cocrystal. Reproduced with permission.^[^ ^130^ ^]^ Copyright 2023, Royal Society of Chemistry.

Given the escalating interest in elucidating the photocatalytic mechanism, the comprehensive understanding of charge separation and transport in multicomponent supramolecular photocatalysts is hindered by the absence of a clear crystal structure and stoichiometric ratio. In this point, our group anchors studies in molecular cocrystals to better understand the behavior of charge separation and transport in multicomponent supramolecular photocatalysts. Molecular cocrystals or organic cocrystals are single crystal materials composed of two or more organic molecules self‐assembled through noncovalent bonds (hydrogen bonds, halogen bonds, π–π interactions, etc.) with good stacking order and well‐defined molecular arrangements,^[^ ^131^ ^]^ a simple representation in **Figure** **13**a. Over the past decade of continuous exploration, molecular cocrystals have demonstrated unique advantages in the fields of nonlinear optics, optical waveguides, luminescence modulation, ambipolar charge transport, and photothermal conversion.^[^ ^57^, ^132^, ^133^, ^134^ ^]^

Like in Figure 13b, we have prepared two molecular cocrystals for photocatalytic removal of environmental pollutants by a simple liquid‐assisted gram‐scale method.^[^ ^130^ ^]^ With the enhanced built‐in electric field between the D‐A molecules by crystal engineering, the charge carriers are separated more easily thermodynamically. The LUMO potential of the cocrystals is elevated owing to the presence of arene‐perfluoroarene (AP) interactions, which leads to an effective enhancement of reduction capacity (Figure 13c). As shown in Figure 13d, the theoretical calculation for the intermolecular pathways of the e^−^ and h^+^ transfer indicates that e^−^ and h^+^ have less chance of encountering each other in cocrystals, which reduces charge recombination. Therefore, this work broadens the study of multicomponent organic supramolecular materials.

### 3.3 Host‐Guest Materials

The host‐guest materials are an essential part of supramolecular chemistry, where the host and guest molecules are identified based on noncovalent bonding interactions such as hydrogen bonding or π–π stacking. Columnar or caged symmetric structures and easy functionalization are usually featured in the host molecules. Also, charge transfer interactions between host and guest aromatic molecules usually expand the absorption of supramolecular materials in the visible region. Together, these characteristics are making host‐guest materials favorable candidates for catalysis, molecular devices, fluorescence sensing, and nonlinear optics.

**Figure 14:**
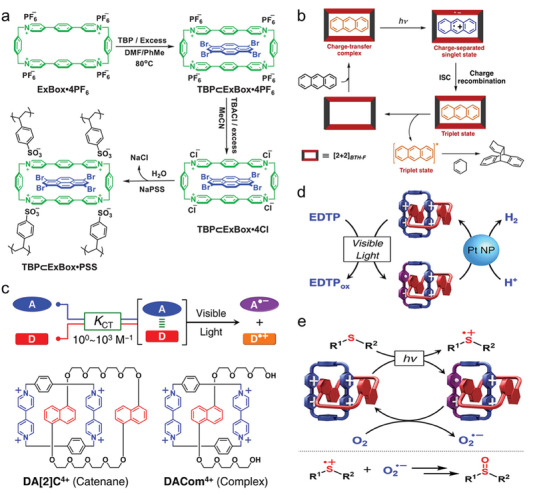
The host‐guest materials for photocatalysis. a) Synthesis process and structure of the TBP⊂ExBox^4+^. Reproduced with permission.^[^ ^135^ ^]^ Copyright 2020, Wiley‐VCH. b) The mechanism of [4+2] cycloaddition in the presence of [2+2]_BTH‐F_. Reproduced with permission.^[^ ^136^ ^]^ Copyright 2020, Wiley‐VCH. c) The mechanism and structure of the D‐A supramolecular complex. d) The mechanism for photocatalytic H_2_ production by using DA[2]C^4+^. e) The mechanism for photocatalytic aerobic oxidation by using DA[2]C^4+^. Reproduced with permission.^[^ ^137^ ^]^ Copyright 2021, American Chemical Society.

The combination of heavy atom spin‐orbit coupling in the 1,3,6,8‐tetrabromopyrene (TBP) guest molecule and photoinduced electron transfer in the TBP⊂ExBox^4+^ supramolecule has been combined by J. Fraser Stoddart et al. to achieve an effective intersystem crossing.^[^ ^135^ ^]^ The homogeneous and inhomogeneous photocatalytic removal of sulfur mustard simulant by TBP⊂ExBox^4+^ is shown to be more effective compared to TBP and ExBox^4+^. As shown in **Figure** **14**a, the combination of large spin‐orbit charge (SOC) of Br atoms and D‐A interaction enhanced spin‐orbit charge transfer intersystem crossing (SOCT‐ISC) enable the single to triplet state (S‐T) transformation efficiently in TBP⊂ExBox^4+^ molecule. The efficient S‐T conversion and internal conversion (IC) relaxation mechanisms are central to the enhancement of ^1^O_2_ generation and increase in photocatalytic performance. Nobuharu Iwasawa et al. have developed a host‐guest photocatalyst anthracene⊂[2+2]_BTH‐F_ by charge transfer excitation of the triplet state in the guest molecule based on the electron‐deficient gust molecule macrocyclic boronic ester [2+2]_BTH‐F_ containing the difluorobenzothiadiazole section (Figure 14b).^[^ ^136^ ^]^ Under visible light excitation, the excited state of anthracene carries out cycloaddition reactions with olefins and several dienes in a [4+2] manner with high yields, and the photocatalyzed cycloaddition reactions are only effective for the included guest molecules. In this work, charge transfer interactions between host‐guest molecules have been used to realize selective catalytic reactions. J. Fraser Stoddart's group has utilized a molecular mechanical interlocking strategy to force the two components of a supramolecular to occupy nanoconfined spaces to enhance CT interactions.^[^ ^137^ ^]^ As in Figure 14c, the D‐A type [2]catenane (DA[2]C^4+^) is formed by electron‐deficient cyclobis(paraquat‐*p*‐phenylene) and electron‐rich 1,5‐dinaphtho[38]crown‐10. Under visible light irradiation, DA[2]C^4+^ can induce the formation of charge‐separated states, which can be converted to DA[2]C^2+^(·+), a persistent free radical species, avoiding the use of other photosensitizers. Compared to the non‐interlocked analog DACom^4+^, DA[2]C^4+^ exhibits enhanced activity in photocatalytic hydrogen production and aerobic oxidation of L‐methionine. This study has proven that D‐A supermolecules are powerful in enhancing charge separation in nanoconfined spaces, as well as providing a new strategy for solar energy conversion and photocatalysis.

### 3.4 Organic Polymer Heterojunctions

Conjugated polymers have undergone significant advancements as photocatalysts, and the D‐A polymer structure has been proven to be one of the key factors in attaining enhanced photocatalytic performance. The incorporation of D‐A mixed heterojunctions has emerged as a highly effective approach for enhancing exciton dissociation, owing to the abundant presence of thousands of D‐A interfaces. These interfaces have been extensively investigated in the context of organic photovoltaics (OPVs).^[^ ^141^, ^142^ ^]^ Recently, polymer bulk heterojunction materials have shown great potential for photocatalysis, and are expected to facilitate the commercial application of organic semiconductor photocatalytic hydrogen evolution.

**Figure 15:**
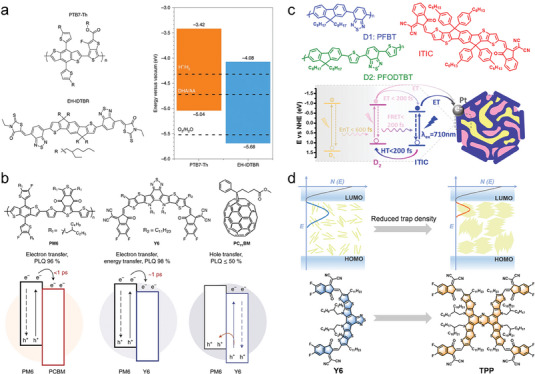
The organic polymer heterojunction materials for photocatalysis. a) Chemical structures and energy levels of PTB7‐Th and EH‐IDTBR. Reproduced with permission.^[^ ^28^ ^]^ Copyright 2020, Springer Nature. b) Chemical structures of PM6, Y6, and PCBM (top), and schematic of exciton decay and electron transfer processes (bottom). Reproduced with permission.^[^ ^138^ ^]^ Copyright 2022, Springer Nature. c) Molecular structures of PFBT (D1), PFODTBT (D2), and ITIC. Reproduced with permission.^[^ ^139^ ^]^ Copyright 2021, American Chemical Society. d) Schematic of low structural disorder and trap density in 2D conjugated materials (top), and chemical structure of TPP. Reproduced with permission.^[^ ^140^ ^]^ Copyright 2021, Wiley‐VCH.

As shown in **Figure** **15**a, it is demonstrated by Iain McCulloch et al. that the generated Z‐type heterojunction between the donor polymer PTB7‐Th and the non‐fullerene acceptor EH‐IDTBR results in a significant enhancement of the photocatalytic hydrogen evolution activity.^[^ ^28^ ^]^ H_2_ release rate (64 426 µmol h^−1^ g^−1^) is increased by order of magnitude when surfactants optimize the heterojunction morphology from an unfavorable core‐shell structure to a tightly packed hybrid D‐A heterojunction. Secondly, the external quantum efficiencies (EQE) exceeded 5% in the visible light range from 660–700 nm. This work has demonstrated the efficiency of solar to chemical energy conversion can be improved by a Z‐type bulk heterojunction scheme. Subsequently, two bulk heterojunction materials PM6:Y6 and PM6:PCBM are synthesized from the donor polymer PBDB‐T‐2F (PM6) matched with the narrow band gap non‐fullerene acceptor BTP‐4F (Y6) or fullerene acceptor PCBM (Figure 15b).^[^ ^138^ ^]^ Under one solar light intensity, PM6:Y6 and PM6:PCBM exhibit hydrogen evolution rates of 43.9 and 73.7 mmol h^−1^ g^−1^, respectively. The effective exciton dissociation in D‐A heterojunctions induces the generation and accumulation of ultralong‐lived photogenerated charges demonstrated by photophysical characterization. Wherein, the photogenerated charge in PM6:Y6 shows more complex behavior, including energy and charge transfer from PM6 to Y6 (Figure 15b bottom), as well as a lower total charge generation efficiency. In contrast, the phase‐separated state in PM6:PCBM nanoparticles further delays the charge complexation, leading to higher EQE and hydrogen evolution performance.

As shown in Figure 15c, Tian et al. have prepared a ternary heterojunction based on a molecule acceptor ITIC and two conjugated polymers (PFBT and PFODTBT) containing benzothiadiazole and fluorene groups.^[^ ^139^ ^]^ Femtosecond transient absorption spectroscopy, steady‐state fluorescence spectroscopy, and spectroelectrochemical measurements are used to reveal the charge transfer and energy transfer processes occurring in ternary and binary polymer systems on sub‐picosecond time scales. Of these, the ITIC molecule is an electron acceptor as well as an energy acceptor. Meanwhile, the crystalline phase of ITIC is formed in the ternary polymer to promote photogenerated electron transferred to Pt cocatalyst. The complete energy and charge transfer pathways between PFBT, PFODTBT, ITIC, and Pt cocatalyst are illustrated in Figure 15c. Lin et al. have designed and synthesized a 2D polycyclic photovoltaic molecule TPP (Figure 15d) for high‐performance photocatalytic hydrogen evolution with an intrinsic trap density of 2.3 × 10^15^ cm^−3^, which is ≈1 to 3 orders of magnitude smaller than typical organic semiconductors.^[^ ^140^ ^]^ TPP molecule possesses ordered molecular stacking, wide light absorption range, and excellent electron mobility. Furthermore, the bulk heterojunction nanoparticles of TPP and PM6 exhibit a photocatalytic hydrogen evolution rate of 72.75 mmol h^−1^ g^−1^, which is superior to the typical PM6:Y6 and even ≈2 to 3 orders of magnitude higher than the inorganic photocatalysts TiO_2_ and CH_3_NH_3_PbI_3_.

As mentioned above, photocatalytic materials based on intermolecular interaction forces are also developing vigorously. Two‐component supramolecules, molecular cocrystals, polymer heterojunctions, and host‐guest materials are often built relying on charge transfer or energy transfer between the donor and acceptor, thus realizing the effect of 1 + 1 > 2. Therefore, researchers are more interested in intermolecular excited state dynamics behaviors to reveal the photophysical behaviors during photocatalysis.^[^ ^143^ ^]^ However, the relevant catalytic active sites have not been fully clarified and validated, making it detrimental to derivative photocatalysts based on intermolecular interactions. Secondly, compared with polymer heterojunctions, the host‐guest and molecular cocrystals have a well‐defined crystal stacking structure, which is a natural advantage for studying the transfer and transport behavior of photogenerated carriers. However, the high crystallinity leads to a generally large particle size, which can affect the photocatalytic performance to a certain extent.^[^ ^14^, ^26^ ^]^ In addition, molecular materials may be attacked by active species, leading to the decomposition of supramolecular interactions and their own structure. Therefore, long‐time testing of photocatalytic activity is an effective method to demonstrate stability.

## 4 Photocatalytic Applications of D‐A Materials

The previous sections emphasize the advantageous properties of D‐A organic photocatalysts, including a broad visible light absorption range, efficient charge separation capability, and low charge recombination. Moreover, their high degree of designability is achieved through the combination of different donor and acceptor molecules, functionalized active centers, and modulation of crystallinity and conjugation degree, which satisfies the demands of photocatalytic applications. Currently, D‐A organic semiconductors are predominantly employed as catalysts in photochemical reactions, such as hydrogen evolution, hydrogen peroxide production, carbon dioxide reduction, pollutant removal, and organic synthesis.

### 4.1 Energy Photocatalysis

#### 4.1.1 Hydrogen Evolution

**Figure 16:**
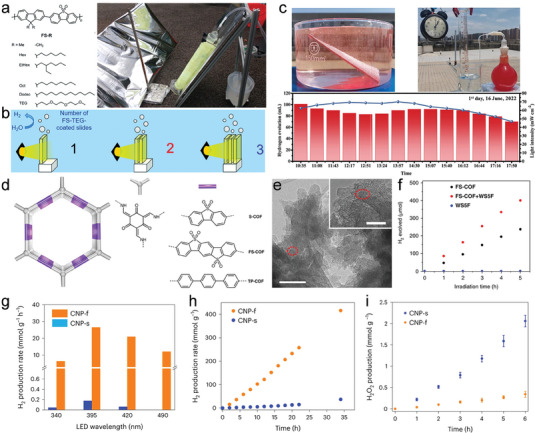
The D‐A type materials for photocatalysis hydrogen evolution. a) The photoreactor set‐up with reflective panel and FS‐TEG‐coated glass fibers. b) Diagram of FS‐TEG‐coated glass slides stacked in series. Reproduced with permission.^[^ ^144^ ^]^ Copyright 2020, Royal Society of Chemistry. c) Hydrogen‐gathering experiments of Py‐TP‐BTDO polymer under natural light. Reproduced with permission.^[^ ^145^ ^]^ Copyright 2023, American Chemical Society. d) Chemical structures of the S‐COF, FS‐COF, and TP‐COF photocatalysts. e) HR‐TEM image of FS‐COF. f) Photocatalytic hydrogen evolution performance of FS‐COF. Reproduced with permission.^[^ ^146^ ^]^ Copyright 2018, Springer Nature. g,h) Photocatalytic hydrogen evolution activities of CNP‐s and CNP‐f. i) Photocatalytic H_2_O_2_ production activities of CNP‐s and CNP‐f. Reproduced with permission.^[^ ^62^ ^]^ Copyright 2023, Springer Nature.

As shown in **Figure** **16**a, Cooper et al. have introduced hydrophilic side chain tri(ethylene glycol) (TEG) into the polymer to increase the swelling of the polymer, which also prolongs the exciton lifetime. As well, dibenzo[*b*, *d*]thiophene sulfone is introduced into the polymer backbone to improve the thermodynamic driving force of photogenerated charge transfer to the sacrificial agent.^[^ ^144^ ^]^ Under visible light irradiation, FS‐TEG powder exhibits a hydrogen evolution rate of 2.9 mmol h^−1^ g^−1^. It is noteworthy that the photocatalytic hydrogen evolution rate of FS‐TEG is as high as 13.9 mmol h^−1^ g^−1^ when it is prepared as a 79 nm thick thin film. In order to verify the photocatalytic performance under natural outdoor conditions, they have designed slides placed in series as shown in Figure 16b to increase the exposure area of the materials. A cylindrical glass reactor is further fabricated using FS‐TEG polymer as a coating for the glass fibers, the hydrogen evolution rate under natural light irradiation is 0.94 L m^−2^ h^−1^.

Subsequently, the research work carried out by Zhang et al. offers more confidence in the practical application of organic photocatalysts. Two types of D‐π‐A CMPs photocatalysts are designed and prepared, employing FSO as the electron acceptor, thiophene as the spacer, and dibenzo[*g*, *p*]chrysene or pyrene as the electron donor. Impressively, large numbers of continuous bubbles (Figure 16c) are rapidly produced and visually observed by the 120 cm^2^ Py‐TP‐BTDO polymer film under natural outdoor sunlight photocatalysis.^[^ ^145^ ^]^ As shown in Figure 16c bottom, outdoor drainage experiments show 1224 mL of hydrogen evolution in 7 h (hydrogen evolution rate up to 312.24 mmol h^−1^ g^−1^), which is one of the highest values for organic photocatalysts so far.

As displayed in Figure 16d, Cooper's group has also reported crystalline FS‐COFs based on a benzo‐bis(benzothiophene sulfone) building block with a much higher photocatalytic hydrogen evolution activity than that of amorphous or semicrystalline polymers.^[^ ^146^ ^]^ Owing to FS‐COF's strong visible light absorption, high crystallinity, and hydrophilic 3.2 nm mesopores (HRTEM image in Figure 16e), it shows a maximum hydrogen evolution rate of 16.3 mmol h^−1^ g^−1^. The colloidal dispersion of FS‐COF is dropped onto a glass carrier to form a COF film, showing a hydrogen evolution rate up to 24.9 mmol h^−1^ m^−2^ after 20 consecutive depositions of the colloidal solution.

As recently reported by Zhu et al, supramolecular material HOF‐H_4_TBAPy shows a photocatalytic hydrogen evolution rate as high as 358 mmol h^−1^ g^−1^, and the apparent quantum yield at 420 nm was 28.6%.^[^ ^14^ ^]^ This further demonstrates the promising application of organic supramolecular materials in the field of photocatalysis. The D‐A structure of the CNP molecule has been prepared by Li et al. and features two distinct aggregate state morphologies (structure in Figure 3b).^[^ ^62^ ^]^ Wherein, the nanofiber (CNP‐f) achieves a hydrogen evolution rate of 31.85 mmol h^−1^ g^−1^ and maintains the stability of the photoreaction for 34 h (Figure 16g–i), which is attributed to the high efficiency of electron transfer from the local CT state to the Pt cocatalyst and intermolecular CT interactions.

The powerful intermolecular built‐in electric field and the extended spectral absorption range are realized due to the variation in D‐A interactions and molecular dipoles between the two component supramolecular materials. As a result, photocatalytic processes such as light absorption, exciton dissociation, and free carrier transport are facilitated. Many excellent studies have been carried out by Zhu's research group to confirm the advantages of two‐component supramolecular materials for photocatalysis. For example, they prepared supramolecular materials such as TCNQ‐PTCDI,^[^ ^147^ ^]^ TPPS/C_60_,^[^ ^127^ ^]^ ZnTCPP/C_60_‐EDA,^[^ ^129^ ^]^ and TPPS/PDI^[^ ^128^ ^]^ for photocatalytic oxygen and hydrogen evolution, and pollutant degradation. Among them, TPPS/PDI shows a great photocatalytic hydrogen evolution rate of 30.36 mmol h^−1^ g^−1^, surpassing most monomolecular materials and D‐A polymers. More details about two component supramolecular materials are shown in Section 3.1 and not repeated here. We summarize the detailed parameters in **Tables** **1** and **2**, considering the large number of D‐A type materials used for photocatalytic hydrogen evolution with different reaction conditions.

**Table 1:** Intermolecular D‐A type materials for photocatalytic hydrogen evolution and the corresponding reaction conditions.

Type	Sample	Light [nm]	Cocatalyst [wt %]	Sacrificial agent	Rate [mmol g^−1^ h^−1^]	Refs.
Supra‐molecule	TPPS/C_60_	Full spectrum	6‐Pt	AA	34.57	[127]
	TPPS/PDI	Full spectrum	Pt	AA	30.36	[128]
	ZnTCPP/C_60_‐EDA	≥ 420	5‐Pt	AA	16.2	[129]
	C_60_@NU‐901	Full spectrum	0.5‐Pt	AA	22.3	[118]
Heterojunction	PM6:TPP	330–1100	20‐Pt	AA	72.75	[140]
	PM6:Y6	330–1100	20‐Pt	AA	62.67	[140]
	PTB7‐Th:EH‐IDTBR	300–800	10‐Pt	AA	60	[28]
	g‐C_3_N_4_/P1Cl‐T	≥ 420	3‐Pt	TEOA	111.8	[35]

**Table 2:** Intramolecular D‐A type materials for photocatalytic hydrogen evolution and the corresponding reaction conditions.

Type	Sample	Light [nm]	Cocatalyst [wt %]	Sacrificial agent	Rate [mmol g^−1^ h^−1^]	Refs.
Small molecule	TPP‐C_60_	≥ 420	3‐Pt	AA	10.69	[67]
	PCPyBDT	≥ 420	Pt	TEOA	0.81	[58]
	EBE	≥ 420	0.04‐Pd	TEOA	0.28	[59]
	CNP‐f	AM 1.5 G	3‐Pt	AA	31.85	[62]
	F1	AM 1.5G	33‐Pt	TEOA‐EG	152.60	[68]
Polymers	DBTD‐CMP1	≥ 420	0.09‐Pd	TEOA	0.92	[72]
	PDBTSO‐T	≥ 300	3‐Pt	AA	147	[71]
	TP‐BTDO‐2	≥ 300	1‐Pt	AA	161.28	[73]
	PyBS‐3	≥ 300	Pt	AA	105	[75]
	Py‐T‐BTDO	≥ 420	1‐Pt	AA	78.4	[148]
	PyTP‐2	≥ 420	None	AA	33.07	[149]
	PyDTDO‐3	≥ 420	None	AA	16.32	[150]
	BTT‐CPP	≥ 300	None	AA	3.79	[76]
	TTB‐CPP	≥ 300	None	AA	0.85	[76]
	Flu‐SO	≥ 420	None	Et_3_N	5.04	[78]
	Py‐SO	≥ 420	None	Et_3_N	4.74	[78]
	PyDF	≥ 420	None	TEOA	18.93	[79]
COFs	NKCOF‐108	≥ 420	5‐Pt	AA	12	[93]
	DABT‐Py	AM 1.5 G	3‐Pt	AA	0.55	[94]
	TAPT‐OMe	≥ 380	2‐Pt	TEA	0.45	[100]
	PyTz‐COF	AM 1.5 G	3‐Pt	AA	0.21	[96]
	g‐C_54_N_6_‐COF	≥ 420	3‐Pt	TEOA	0.25	[102]
	COF‐JLU35	420–780	1‐Pt	AA	70.8	[104]
	COF‐JLU36	420–780	1‐Pt	AA	23.6	[104]
	TtaTfa	≥ 420	8‐Pt	AA	20.7	[151]

#### 4.1.2 Hydrogen Peroxide Production

Hydrogen peroxide (H_2_O_2_) is an essential green chemical substance that shows tremendous potential for applications in the paper industry, disinfection, wastewater treatment, and fuel cells.^[^ ^156^ ^]^ Anthraquinone and catalytic oxidation of hydrogen gas methods are typically used for industrial‐scale production of H_2_O_2_, and both methods have serious shortcomings which are related to energy losses, environmental pollution, and complicated process steps. As a green proposal to utilize renewable solar energy, photocatalytic H_2_O_2_ generation at low concentrations is gradually attracting attention.^[^ ^157^ ^]^ Recent studies indicate that, compared to noble metal photocatalysts and inorganic materials, organic semiconductors often demonstrate enhanced activity and selectivity in the photocatalytic production of H_2_O_2_. This can be attributed to their ability to form appropriate intermediates and effectively eliminate undesired side reactions.

**Figure 17:**
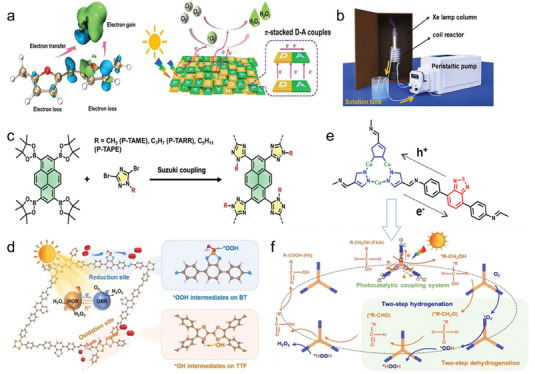
The D‐A type materials for photocatalysis H_2_O_2_ generation. a) Charge difference map of D‐A furan couple system, and schematic of H_2_O_2_ production mechanism. b) Scaled‐up photocatalytic system produced H_2_O_2_ continuously for 24 h. Reproduced with permission.^[^ ^152^ ^]^ Copyright 2023, Elsevier. c) The synthetic routes for the TA‐based polymer photocatalysts. Reproduced with permission.^[^ ^153^ ^]^ Copyright 2023, Wiley‐VCH. d) Schematic of the oxidation‐reduction molecular junction COF photocatalyst. Reproduced with permission.^[^ ^154^ ^]^ Copyright 2023, Wiley‐VCH. e) The chemical structure of Cu_3_‐BT‐COF. f) Schematic of mechanism for H_2_O_2_ photosynthesis coupled with FAA photo‐oxidation by Cu_3_‐BT‐COF. Reproduced with permission.^[^ ^155^ ^]^ Copyright 2023, Wiley‐VCH.

Hydrothermal carbons (HTCs) with new furan‐resin‐structures containing conjugated quinoid and aromatic furan units for photocatalytic H_2_O_2_ production are reported by Mao et al. The photocatalytic H_2_O_2_ production rate of HTCs reached 480.7 µmol h^−1^ g^−1^ without sacrificial agent and O_2_ aeration.^[^ ^152^ ^]^ As shown in **Figure** **17**a, a significant increase in the photogenerated charge transfer efficiency is achieved by the π stacked D‐A units in the main chain of the furan resin, which is the main reason for the high activity. In addition, the HTCs realize continuous H_2_O_2_ production for 24 h in the scaled‐up spiral photocatalytic flow reactor (Figure 17b), and the final H_2_O_2_ enriched concentration is 2.464 mM. Wang et al. have introduced electron‐deficient triazole (TA) units and electron‐rich Py units (Figure 17c) into the polymer backbone to prepare a series of D‐A polymers with different alkyl chain lengths.^[^ ^153^ ^]^ In contrast, P‐TAME (with methyl side chains) shows the strongest D‐A interactions, the smallest exciton binding energy, and good hydrophilicity, and is found to exhibit the H_2_O_2_ production rate of 9.5 mmol h^−1^ g^−1^ under visible light irradiation (O_2_ aeration, phenylcarbinol as sacrificial agent), which is superior to most polymers reported to date.

**Figure 18:**
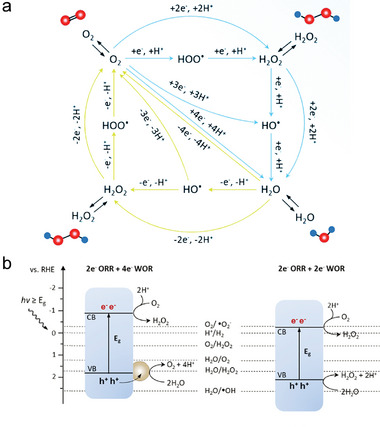
Electronic reactions and energy band structures involved in the photosynthesis of H_2_O_2_. a) The oxidation or reduction pathway for the photosynthesis of H_2_O_2_ from H_2_O and O_2_. b) Different redox pathways for photosynthesis H_2_O_2_ formation, and the corresponding redox potential. Reproduced with permission.^[^ ^158^ ^]^ Copyright 2020, Royal Society of Chemistry.

The most optimal approach for the synthesis of H_2_O_2_ is through the utilization of full reaction photosynthesis, wherein the concurrent processes of water oxidation (WOR) and oxygen reduction (ORR) occur without the requirement of sacrificial agents, thereby maximizing energy efficiency. In contrast to the two‐electron ORR pathway, WOR encompasses three competing routes (**Figure** **18**a), including the four‐electron transfer pathway for photoinduced oxygen evolution, the two‐electron transfer pathway for H_2_O_2_ production, and the single‐electron transfer pathway for the generation of hydroxyl radical (·OH).^[^ ^158^ ^]^ Consequently, enhancing the efficiency of the two‐electron WOR pathway assumes utmost importance in achieving heightened efficacy in H_2_O_2_ synthesis through photosynthetic means. However, as shown in Figure 18b, because the two‐electron WOR is a thermodynamic uphill reaction (1.76 V versus normalized hydrogen electrode), this is difficult to achieve in general.^[^ ^159^ ^]^

By considering the structural tunability of COFs and the endowed ligand‐to‐ligand charge transfer effect, Xu et al. have achieved efficient photocatalytic production of H_2_O_2_ via the WOR and ORR pathways employing acetylene‐containing CTFs as the active material. In CTF‐BDDBN material,^[^ ^106^ ^]^ the solar chemical conversion efficiency (SCC) reaches 0.14% and with 100% atom utilization efficiency. As shown in Figure 17d, a D‐A type COF with oxidation and reduction centers (TTF‐BT‐COF), in which benzothiazole (BT) serves as the reduction site and tetrathiafulvalene (TTF) as the oxidation site, is prepared for the first time by Lan et al. In the absence of a sacrificial agent, TTF‐BT‐COF exhibits a record photocatalytic H_2_O_2_ production rate, as high as 276 000 µM h^−1^ g^−1^.^[^ ^154^ ^]^ The excellent photocatalytic performance is attributed to the effective separation of h^+^ and e^−^ due to the D‐A interaction in TTF‐BT‐COF and allows the e^−^ and h^+^ to carry out the redox reactions at the BT site and TTF site, respectively. Characterization by EPR, in situ diffuse reflectance infrared spectroscopy (DRIFTs), ^18^O_2_ isotope experiments, rotating disk electrode (RDE), and rotating ring disk electrode (RRDE) verify the simultaneous two‐electron ORR and two‐electron WOR reactions in TTF‐BT‐COF from various aspects. Their group has obtained a series of oxidation‐reduction molecular junctions COFs with different functional groups through covalent linkages between metal clusters and organic molecules. As shown in Figure 17e, the most optimal efficiencies in the photo‐oxidation of furfuryl alcohol (FFA) and H_2_O_2_ photosynthesis coupling reaction are achieved in Cu_3_‐BT‐COF, which is constructed by covalently linking Cu_3_ metal clusters with thiazole (BT).^[^ ^155^ ^]^ Theoretical calculations have demonstrated that the reaction substrates FAA and O_2_ are easier to adsorb onto Cu_3_ and BT, respectively. Photogenerated electrons can be more efficiently transferred from Cu_3_ to the BT to generate e^−^ and h^+^ for H_2_O_2_ photosynthesis and FFA photo‐oxidation reactions, thus enabling coupled reactions. Figure 17f shows the complete mechanism of the synergistic hydrogenation and dehydrogenation of Cu_3_‐BT‐COF on photosynthesis of H_2_O_2_ with FAA photo‐oxidation, with a clear presentation of the catalytic reaction sites and the associated redox processes.

#### 4.1.3 Carbon Dioxide Reduction

The excessive consumption of fossil fuels by human activities has resulted in unrestricted emissions of carbon dioxide (CO_2_) into the atmosphere. This phenomenon has given rise to numerous global challenges, including energy crises and the adverse impacts of global warming. Addressing these issues necessitates the development of sustainable methods that leverage solar energy to mitigate atmospheric CO_2_ levels while simultaneously converting it into high‐value chemical products.^[^ ^162^ ^]^ Although substantial progress has been made in the field of photocatalysts, research focused on the photocatalytic reduction of CO_2_ is still at an early stage. In this section, we provide a concise overview of organic photocatalysts with a D‐A architecture, showcasing their exceptional performance in facilitating the photocatalytic reduction of CO_2_.

**Figure 19:**
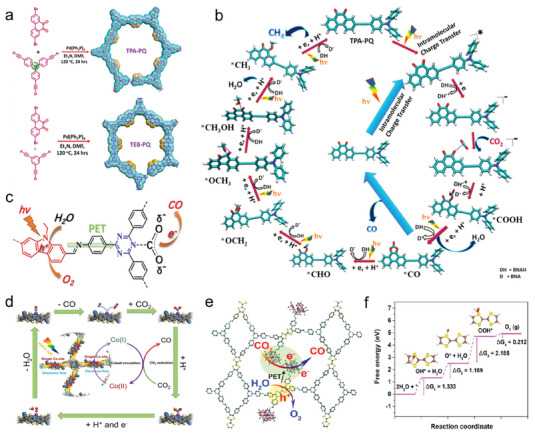
The D‐A type materials for photocatalysis CO_2_ reduction. a) Schematic for the structure and synthesis of TPA‐PQ. b) Catalytic cycle with the different intermediates. Reproduced with permission.^[^ ^160^ ^]^ Copyright 2021, American Chemical Society. c) Electron transfer and reaction pathway in CT‐COF for the photoreduction of CO_2_. Reproduced with permission.^[^ ^161^ ^]^ Copyright 2020, Wiley‐VCH. d) The mechanism of sp^2^c‐COF_dpy_‐Co for CO_2_ photoreduction reaction. Reproduced with permission.^[^ ^121^ ^]^ Copyright 2020, Elsevier. e) The mechanism of TCOF‐MnMo_6_ for H_2_O oxidation and CO_2_RR. f) Free energy for H_2_O oxidation in TCOF‐MnMo_6_. Reproduced with permission.^[^ ^120^ ^]^ Copyright 2022, American Chemical Society.

The tunable band gap, high CO_2_ adsorption capacity, and abundant active sites make organic porous materials promising candidates for photocatalytic CO_2_ reduction. Tapas Kumar Maji et al. have developed a redox‐active CMPs (TPA‐PQ) by merging the donor tris(4‐ethynylphenyl)amine (TPA), and the acceptor phenanthraquinone (PQ), the structure is displayed in **Figure** **19**a.^[^ ^160^ ^]^ As a metal‐free multiphase photocatalyst for visible‐light‐driven CO_2_ reduction to CH_4_, TPA‐PQ exhibits an impressive rate (2.15 mmol h^−1^ g^−1^) and high selectivity (> 97%). The internal charge separation (ICT) from TPA to PQ is shown due to the strong D‐A property in TPA‐PQ, which induces electron enrichment on the PQ unit to reduce CO_2_ adsorbed on the keto group and reduces the e^−^‐h^+^ pair recombination. As shown in Figure 19b, in situ DRIFTS combined with theoretical calculations have revealed the conversion of *COOH intermediates to *CO via protonation and water elimination. The high stability of *CO hinders the desorption of CO, thus CO_2_ is reduced to CO with low efficiency. Subsequently, *CO undergoes a multi‐step intermediate transformation to CH_4_ with the participation of the sacrificial agent triethylamine (TEA) and 1‐benzyl‐1,4‐dihydronicotinamide (BNAH). Visible light‐driven reduction of CO_2_ to valuable chemicals without sacrificial agents and co‐catalysts remains challenging. The highly crystalline D‐A type COFs (CT‐COFs) of the carbazole‐triazine group have been constructed via Schiff base reaction by Kong et al. CO_2_ is reduced to CO and water is oxidized to produce O_2_ with approximate stoichiometry.^[^ ^161^ ^]^ As shown in Figure 19c, the authors have proposed that when N1, which is the CO_2_ adsorption and electron export site, is conjugated to LUMO, the photogenerated e^−^ migrate from the HOMO of the carbazole to N1 via ICT, and then are transferred to the adsorbed CO_2_ for the reaction. Photogenerated h^+^ in the carbazole fraction reacts with H_2_O to precipitate O_2_. Overall, this research work has demonstrated a good example of using a metal‐free photocatalytic system to achieve the overall conversion of CO_2_ and H_2_O to CO and O_2_.

Wang et al. have synthesized the Co‐metal modified D‐A type COF (sp^2^c‐COF_dpy_‐Co) showing high selectivity for photocatalytic reduction of CO_2_ to CO.^[^ ^121^ ^]^ The high selectivity is attributed to the Co sites facilitating electron transport from the nitrogen atom to the chelating metal, serving as a bridge to inject electrons into the CO_2_ molecule. The whole sequence is displayed schematically in Figure 19d, where the Co (II) sites maintain the cycling of Co (II) and Co(I) during the reaction process, and support the reduction of CO_2_ to CO via the proton‐electron coupling method. The metal clusters existing in the D‐A type COF (TCOF‐MnMo_6_) also play the role of active sites in photocatalytic CO_2_ reduction. As shown in Figure 19e, under visible light irradiation, the photogenerated e^−^ is transferred from the TTF portion (HOMO centers) to the MnMo_6_ portion (LUMO centers) and thus to the active sites in MnMo_6_ for CO_2_ reduction.^[^ ^120^ ^]^ At the same time, the photogenerated h^+^ in TTF are able to oxidize H_2_O to O_2_, and Figure 19f demonstrates the free energy diagram of the oxidation process in TCOF‐MnMo_6_. This work has systematically explained the active site and reaction thermodynamics of CO_2_ reduction and H_2_O oxidation in a meaningful way.

To date, little organic small molecule materials are reported for photocatalytic CO_2_ reduction. Wang et al. have documented the utilization of fulleropyrrolidine functionalized with 4,7‐di(thiophen‐2‐yl)benzo[*c*][1,2,5]thiadiazole (DTBT),^[^ ^163^ ^]^ demonstrating its good efficacy in facilitating the photochemical reduction of CO_2_ to CO. The covalent bonding between the DTBT moiety and C_60_ molecules is found to exert a pronounced impact, considerably augmenting the rate of exciton generation, and effectively extending the longevity of charge carriers. A series of small D‐A type molecules based on thiophene and NDI units are also synthesized by their group and used for photocatalytic CO_2_ reduction,^[^ ^60^ ^]^ and the molecular structures have been shown in Figure 2c. Among them, with the appropriate redox potential, the photocatalytic CO_2_ to CO conversion yield of NDI‐4T is 168.6 µmol g^−1^ 24 h^−1^ (Figure 19g). Though it does not show surprising photocatalytic performance compared to polymers, it also demonstrates the potential that D‐A molecules can be used for photocatalytic CO_2_ conversion. Therefore, organic small molecule materials for high‐performance photocatalytic CO_2_ reduction urgently need to be developed by researchers to achieve in‐depth knowledge and breakthroughs in the mechanism.

### 4.2 Environmental Photocatalysis

Photocatalytic degradation of environmental pollutants is emerging as a prospective strategy for mitigating various environmental crises. It offers several advantages, such as simplicity of operation, mild reaction conditions, and minimal secondary pollution.^[^ ^164^ ^]^ To be effective in environmental photocatalysis, materials should possess excellent surface adsorption capacity, suitable band structure, outstanding chemical stability, and be easily scalable. Organic semiconductors, which utilize abundant elements on earth, exhibit favorable characteristics in heterogeneous catalysis. They demonstrate ease of separation and cycling stability, making them a promising material for addressing environmental challenges.^[^ ^165^ ^]^

**Figure 20:**
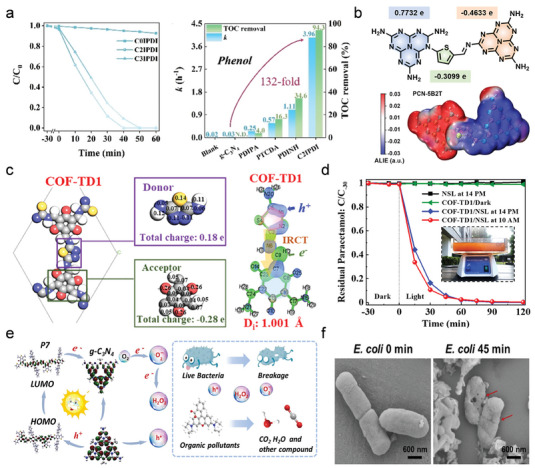
The D‐A type materials for environmental photocatalysis. a) Photocatalytic phenol degradation performance of PDI derivatives. Reproduced with permission.^[^ ^63^ ^]^ Copyright 2023, Wiley‐VCH. b) The structure and electrostatic potential distribution of PCN‐5B2T. Reproduced with permission.^[^ ^166^ ^]^ Copyright 2023, American Chemical Society. c) Chemical and electronic structure of COF‐TD1. d) Photocatalytic degradation performance of COF‐TD1 in a scale‐up reactor under natural sunlight irradiation. Reproduced with permission.^[^ ^167^ ^]^ Copyright 2022, American Chemical Society. e) The diagram of the photocatalytic sterilization mechanism of P7/g‐C_3_N_4_. f) SEM images of *E. coli* before and after treatment with P7/g‐C_3_N_4_. Reproduced with permission.^[^ ^168^ ^]^ Copyright 2022, Elsevier.

Chen et al. have investigated the influence of carbon chain length on the π‐π stacking distance and molecular packing patterns of PDI‐derived molecules.^[^ ^63^ ^]^ Notably, C2IPDI molecules containing ethyl groups exhibit the shortest π–π stacking distance and the fastest charge carrier transport, the structure of C2IPDI is shown in Figure 4a. As a result (**Figure** **20**a), phenol is fully degraded by C2IPDI in 35 min and the total organic carbon removal (TOC) is over 94%, about 32 times higher than that of C0IPDI without σ bridges. Compared with other reported PDI‐based organic photocatalysts and g‐C_3_N_4_, C2IPDI exhibits the highest efficiency in photocatalytic degradation of phenol and TOC removal. It is well known that bromate is classified as a potential carcinogen by the International Agency for Research on Cancer (IARC) for increasing the risk of cancer in humans and is harmful to human health. The D‐A molecular photocatalyst BDTD composed of an aldehyde‐containing benzene and triphenylamine has been synthesized by You et al. to degrade bromate to bromide by almost 100%, showing excellent photocatalytic bromate reduction ability.^[^ ^65^ ^]^

The carbon nitride‐based (PCN) D‐π‐A polymer PCN‐5B2T has been constructed by Ao et al. through copolymerization of urea with 5‐bromo‐2‐thiophenecarboxaldehyde, and the structure is demonstrated in Figure 20b. The abundance of micro‐, meso‐, and macropores in PCN‐5B2T greatly facilitates the adsorption of pollutants and intramolecular charge transfer, resulting in a nearly 10‐fold increase in the photocatalytic degradation rate of 2‐mercaptobenzothiazole (2‐MBT).^[^ ^166^ ^]^ Theoretical calculations indicate that the 2‐MBT molecules are more easily adsorbed on the π bridges and oxidized with h^+^, and the e^−^ are more readily transferred from the D unit (tertiary amine group) to the acceptor imine unit via the thiophene π bridges, which enable an efficient e^−^‐h^+^ separation. As shown in Figure 20c, two D‐A characterized COF photocatalysts, COF‐TD1 and COF‐TD2,^[^ ^167^ ^]^ have been prepared by integrating the 1,3,4‐thiadiazole or 1,2,4‐thiadiazole ring (D units) and the quinone (A units) into the COF skeleton by Tong et al. Electrons are more easily excited in the 1,3,4‐thiadiazole molecule, and COF‐TD1 yields excellent photodegradation properties, degrading > 98% of paracetamol in 60 min. In the scaled‐up experiments in the natural environment, COF‐TD1 still maintains the photocatalytic performance the same as in the laboratory (Figure 20d). In addition, COF‐TD1 exhibits the excellent photocatalytic degradation of paracetamol in both river and lake water environments, as well as the universal degradation of bisphenol A, naproxen, and diclofenac.

Constructing polymer heterojunctions has become a pervasive solution in the pursuit of superior photocatalytic sterilization and pollutant degradation performance. Four D‐A conjugated polymers (P4, P5, P7, and P9) have been synthesized by Sun et al. based on benzodithiophene (BDT) containing carbazole side chains. Then, carbon nitride‐based polymer heterojunctions for photocatalytic sterilization and pollutant degradation are successfully constructed. As shown in Figure 20e, the balance of light absorption, charge separation efficiency, and energy band structure are achieved in the P7/g‐C_3_N_4_ heterojunction, with optimal photocatalytic sterilization and pollutant degradation performance.^[^ ^168^ ^]^ The inactivation of *Escherichia coli* and *Staphylococcus aureus* by P7/g‐C_3_N_4_ is more than 99.7% after 45 and 90 min of light irradiation, and the SEM pictures in Figure 20f also indicate the destruction of *E. coli*.

### 4.3 Organic Synthesis

Due to its mild reaction conditions and the ability to harness solar energy, photocatalytic organic synthesis is considered a promising green synthetic approach. By modulating the D‐A interactions, the band positions and band gap can be adjusted, leading to enhanced catalytic selectivity. Additionally, D‐A interactions can improve charge separation efficiency and significantly enhance catalytic performance, making D‐A type organic semiconductors frequently employed as heterogeneous catalysts for photocatalytic organic synthesis.

**Figure 21:**
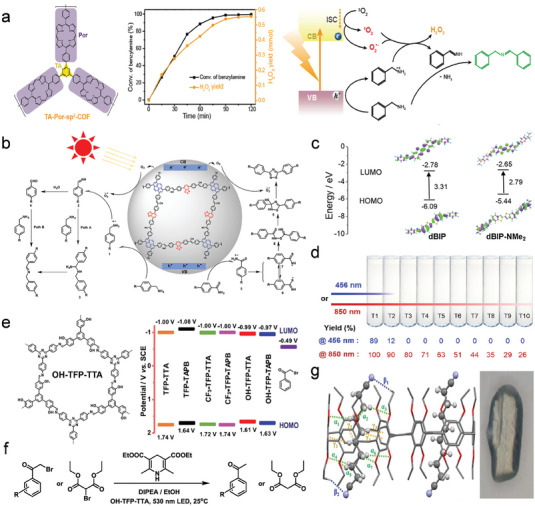
The D‐A type materials for organic transformations. a) The structure of TA‐Por‐sp^2^‐COF, and the reaction path of the benzylamine coupling. Reproduced with permission.^[^ ^169^ ^]^ Copyright 2022, American Chemical Society. b) Photocatalytic mechanisms of Py‐BSZ‐COF‐mediated oxidative amine coupling. Reproduced with permission.^[^ ^103^ ^]^ Copyright 2020, American Chemical Society. c) The energy level of the dBIP molecule with two‐photon absorption properties. d) Photocatalytic performance of dBIP under infrared irradiation. Reproduced with permission.^[^ ^170^ ^]^ Copyright 2023, American Chemical Society. e) The structure of D‐A type COF OH‐TFP‐TTA, and schematic energy band structure with different compositions. f) The photo‐reductive dehalogenation reactions over OH‐TFP‐TTA. Reproduced with permission.^[^ ^171^ ^]^ Copyright 2020, American Chemical Society. g) The crystal structure and pictures of crystals of *m*‐TPE Di‐EtP5⊃G2. Reproduced with permission.^[^ ^172^ ^]^ Copyright 2023, Wiley‐VCH.

D‐A‐based materials are widely used for photocatalytic oxidative amine coupling reactions. As shown in **Figure** **21**a, a new hyper‐conjugated COF (TA‐Por‐sp^2^‐COF) has been synthesized by Yu et al. using a cyano group‐terminated porphyrin as a precursor through the self‐polymerization of cyano groups.^[^ ^169^ ^]^ The TA‐Por‐sp^2^‐COF demonstrates excellent photocatalytic performance in the photocatalytic thioanisole selective oxidation and coupling reaction of benzylamine due to the synergistic effect of D‐A fragments in inducing photogenerated carrier separation and the large porosity for diffusion. Benzylamine can be completely converted within 2 h and the conversion and selectivity are above 99%, accompanied by the production of H_2_O_2_ (0.556 mmol). In addition, an excellent catalytic effect of TA‐Por‐sp^2^‐COF has been achieved for all benzylamine derivatives, with over 95% conversion in 3 h, and the selectivity remains above 99%. As in Figure 21b, Li et al. have designed and synthesized a D‐A type COF (Py‐BSZ‐COF) containing Py units and BT units.^[^ ^103^ ^]^ Under visible light irradiation, ·O_2_^−^ is generated from Py‐BSZ‐COF in air‐saturated acetonitrile, which drives photocatalytic oxidative amine coupling in good yields and high recoverability. In addition, experimental results under mild irradiation conditions demonstrate that a variety of thioamides could be converted to 1,2,4‐thiadiazole in good yields.

Sun et al. have utilized the two‐photon absorption (TPA) property of organic molecules to cleverly drive various organic reactions involving O_2_ using near‐infrared light at 850 nm and show excellent performance.^[^ ^170^ ^]^ As shown in Figure 21c, the dBIP molecule consists of the bibenzothiazole as the electron acceptor and the two benzene rings connected by imine bonds as the electron donors. They compare the photocatalytic performance of dBIP for benzylamine coupling under the irradiation of 850 and 456 nm light sources, only the light source is allowed to excite the reaction tube from the left side (Figure 21d). Under irradiation at 456 nm, 89% yield is achieved in the first reactor tube, while the second reactor tube has only 12% yield, and the remaining few reactors have a conversion performance of 0%. It is exciting to note that 850 nm irradiation exhibited 100% yield not only in the first reaction tube, but also attractive catalytic yields in the second (90%), third (80%), and fourth (71%) tubes. In summary, this work emphasizes the utilization of TPA properties for the development of molecular photocatalysts that operate in the near‐infrared region.

Photocatalytic dehalogenation of *α*‐bromoacetophenone is also widely studied. Li et al. have synthesized multiple [3+3] COFs with 2D hexagonal pore structures for photocatalytic reductive dehalogenation reactions. The substituents are used for bandgap tuning (Figure 21e), where COF containing the –OH substituent has the narrowest bandgap and the strongest absorption of visible light.^[^ ^171^ ^]^ As in Figure 21f, OH‐TFP‐TTA with both D‐A structure and –OH exhibits the highest photocatalytic reductive dehalogenation activity. The D‐A structure of COF‐JLU22 has been synthesized by Liu et al. and displays a large specific surface area, excellent photoelectric response, and broad visible light absorption.^[^ ^173^ ^]^ Under visible light excitation, COF‐JLU22 shows good conversion efficiency and selectivity for reductive dehalogenation of phenacyl bromide derivatives and *α*‐alkylation of aldehydes.

Hu et al. have synthesized dimeric fluorescent macrocyclic *m*‐TPE Di‐EtP5 (meso‐tetraphenylethylene dimeric ethoxypillar[5]arene) containing both two pillar[5]arenes and tetraphenylene (TPE) components.^[^ ^172^ ^]^ Three dinitrile derivatives with different structures are designed as guests (G1, G2, and G3) to form the host‐guest supramolecule with *m*‐TPE Di‐EtP5. Among them, G3 has a bulky phenyl group in the middle of the molecular chain, which is further assembled with *m*‐TPE Di‐EtP5 to precipitate as supramolecular polymer powder (a structure analogous to that of *m*‐TPE Di‐EtP5⊃G2, Figure 21g). When *m*‐TPE Di‐EtP5⊃G3 is used as a photocatalyst, 2‐bromo‐1‐phenylethanone is irradiated with white light for 2 h to obtain the product acetophenone at a conversion rate of 45%, which is further increased to 99% upon binding to the acceptor eosin Y.

Photocatalysis is widely used in various fields covering chemistry, energy, environment, and health of life. Many studies have been carried out to realize photocatalytic hydrogen evolution under real sunlight irradiation, mainly focusing on polymers and COFs, but also a few supramolecular materials with such properties.^[^ ^174^ ^]^ But sacrificial agents are still required to participate in the reaction to achieve overall water splitting in fewer examples. At the same time, because of the low efficiency of photocatalysis, the hydrogen evolution efficiency needs to be improved by the involvement of other approaches, such as photovoltaic‐electrolytic water splitting. Photocatalytic hydrogen peroxide production can be achieved without adding any sacrificial agent and can utilize both electrons and holes, achieving theoretically 100% exciton utilization. The industrial preparation of H_2_O_2_ is more concentrated and the process is mature, and photocatalytic technology can be used as a complement to achieve small‐scale, decentralized H_2_O_2_ preparation. The conversion of CO_2_ into value‐added products has received a lot of attention, but the photocatalytic CO_2_ reduction reaction is a multi‐electron transfer process with a wide variety of products. Therefore, the materials are required to have both CO_2_ adsorption, excellent reduction, and selectivity, making the photocatalytic materials more demanding.^[^ ^175^ ^]^ The involvement of metal co‐catalysts is usually also required to promote highly active and selective conversion of CO_2_. For photocatalytic organic synthesis, environmental pollutant degradation, and sterilization, the active species produced by photocatalysis, such as hydroxyl radicals, oxidizing properties of superoxide radicals, and selective oxidation of singlet oxygen, are usually utilized. These active species can affect the bonding of target molecules and the cellular structure of bacteria, culminating in substance synthesis, degradation, and bactericidal functions. Also, the photocatalytic material should have good adsorption of the substrate molecules and desorption of the target products so that the reactions go forward.^[^ ^176^ ^]^

From the thermodynamic point of view, whether a certain oxidation or reduction reaction can take place or not is dependent on the valence band (VB) and conduction band (CB) potentials of the materials. While satisfying thermodynamics, the kinetic behavior of semiconductor materials tends to affect the efficient conduct of photocatalytic reactions.^[^ ^177^ ^]^ These involve abundant and complex physicochemical processes such as energy band structure, charge transport, charge recombination, surface reaction, active intermediate, spatial mass transfer, and so on. In terms of thermodynamics, D‐A type organic photocatalytic materials tend to broaden the light absorption range and modulate the redox potential, better fitting the photocatalytic reaction.^[^ ^178^ ^]^ In dynamics, the D‐A interactions are beneficial for the construction of a built‐in electric field, thus reducing the charge transfer resistance and inhibiting charge recombination. At the same time, the D‐A structure is beneficial for prolonging the charge‐separated state lifetime and increasing the chances of photocatalytic reactions.^[^ ^179^ ^]^ Of course, a series of assistant strategies, such as porous materials, crystal engineering, and interface engineering, can further enhance the performance of D‐A organic photocatalytic materials.^[^ ^180^ ^]^

On the whole, the efficiency of photocatalysis is generally lower now, and there is still a long distance to industrial application, which requires researchers to keep exploring in structure and process of photocatalytic materials.

## 5 Summary and Outlook

Strongly bound excitons and low mobility charge carriers are generated in organic semiconductors upon photoexcitation, which directly limits charge separation and transport in organic materials and ultimately limits photocatalytic performance. However, these intrinsic shortcomings can be effectively mitigated due to the convenient solution‐processability and easy chemical functionalization of organic semiconductors, allowing for customizing the structure and function of molecules, supramolecules, and polymers. In this paper, we review the modification strategies for organic semiconductor materials first. Subsequently, we discuss organic semiconductor materials with high photocatalytic activity from the perspective of donor‐acceptor interactions, including intramolecular D‐A interactions and intermolecular D‐A interactions. Meanwhile, from the crystallinity aspect, we review supramolecular, polymeric, and crystalline polymeric photocatalysts with D‐A structures. Finally, the applications of various types of D‐A materials in the fields of energy photocatalysis (H_2_ evolution, H_2_O_2_ production, and CO_2_ reduction), environmental pollutant removal, and organic synthesis are briefly summarized.

Overall, D‐A type organic semiconductor materials have been widely studied and applied on a scale in photocatalysis due to their good visible light absorption, high exciton dissociation efficiency, long carrier lifetime, and a large degree of π‐electron delocalization. The choice of different bonding modes, the embedding of functional groups, and the combination of various donors and acceptors lead to a high degree of structural tunability and controllability of the photoelectric function of the D‐A type materials. However, there are still some challenges and difficulties that need to be solved in the studies of D‐A type organic photocatalytic materials.

1) In comparison to polymeric photocatalytic materials, D‐A molecular materials are less well studied. The synthesized D‐A molecules or supramolecules normally employ one type of A unit paired with one type of D unit, resulting in a relatively small degree of π‐conjugation, which affects the performance enhancement to a certain extent. Learning from the development of polymers, designing molecular materials with multiple donor‐acceptor fragments, such as A1‐D‐A2 and D1‐A‐D2, might lead to different photophysical‐chemical properties. As can be expected, this will significantly increase the difficulty and time cost of the material synthesis process.
2) Both conjugated polymers and COFs exhibit large conjugation areas and stable bonding modes, and it is very beneficial for increasing photocatalytic performance. However, the poorer crystallinity and amorphous topology are unfavorable for the transport of photogenerated carriers, and the probability of carriers participating in surface‐catalyzed reactions is reduced. Therefore, the preparation of highly crystalline and long‐range ordered polymeric materials via spatially restricted domains, interface growth, template methods and other nucleation, and crystallization strategies are desirable.
3) More and more heterojunction and hybridized organic semiconductor materials are applied to photocatalytic reactions. However, the process of diffusion, separation, and transport of excitons in organic semiconductor interfaces is still highly controversial considering the complexity of the electronic structure of the material interface and the roughness of the characterization tools. At the same time, a series of physicochemical issues such as charge migration, catalytic substrate conversion, and product release in the interface between the material and the reacting substrate contact are also in urgent need of clarification. As such, utilizing more accurate in situ testing, advanced computation, and theoretical simulations at the atomic level will offer more opportunities for interface optimization.
4) Whether conjugated microporous polymers, COFs, heterojunctions, or supramolecular materials, the design and synthesis of D‐A photocatalytic materials are mostly based on previous experiences. Reliable theoretical foundations are lacking for the selection of the pairing components between D and A and for the determination of the mode of attachment (covalent or noncovalent bonding). With the spread of information technology and the refinement of artificial intelligence, the introduction of machine learning in the design of D‐A materials to predict the intrinsic properties and photocatalytic performance may have realized the early application of photocatalytic technology.

The customization of D‐A type organic photocatalytic materials is a difficult development path, although lots of experimental results have proven its promising development. But we believe that with the continuous progress of the theoretical level, synthesis technology and the continuous efforts of researchers, organic semiconductor photocatalytic materials will be brilliant in basic research and industrialized applications.

## Conflict of Interest

The authors declare no conflict of interest.
